# OsNTL2 confers rice osmotic stress resilience through coordinated transcriptional regulation of the ASC-GSH redox cycle and cell wall biosynthesis

**DOI:** 10.1007/s44154-025-00271-4

**Published:** 2025-11-27

**Authors:** Heng Zhou, Xiaoyun Ma, Jianping Yang, Lingxi Geng, Taotao Qiu, Xinyue Fan, Kailu Zhang, Fuyuan Zhu, Yanjie Xie

**Affiliations:** 1 Laboratory Center of Life Sciences, College of Life Sciences, Nanjing Agricultural University, Nanjing, 210095 China; 2 National Key Laboratory for the Development and Utilization of Forest Food Resources, Co-Innovation Center for Sustainable Forestry in Southern China, State Key Laboratory of Tree Genetics and Breeding, Key Laboratory of State Forestry and Grassland Administration On Subtropical Forest Biodiversity Conservation, College of Life Sciences, Nanjing Forestry University, Nanjing, 210037 Jiangsu China

**Keywords:** NAC TF, Osmotic stress, ASC-GSH cycle, Transcriptional regulation, Cell wall

## Abstract

Drought, high salinity, and low temperatures impose osmotic stress, hindering water uptake and severely limiting plant growth and crop productivity. Osmotic stress not only perturbs cellular osmotic homeostasis but also disrupts multiple metabolic processes, including reactive oxygen species (ROS) metabolism. However, the transcriptional regulation underlying these redox processes in plants remains poorly understood. Here, we report that rice NAC WITH TRANS-MEMBRANE MOTIF1- LIKE 2 (OsNTL2) is required for tolerance to salt and osmotic stresses. DNA affinity purification-sequencing (DAP-seq) revealed that OsNTL2 directly targets key genes in the ascorbate–glutathione (ASC-GSH) redox cycle, including *ascorbate peroxidase 2* (*APX2*), *monodehydroascorbate reductase 1* (*MDHAR1*), *glutathione reductase 2* (*GR2*), and *glutathione peroxidase 5* (*GPX5*), as well as *peroxidase 3/70* (*PRX3/70*), which function in the hydrogen peroxide catabolic process. Consistently, OsNTL2 activity was associated with enhanced ASC-GSH cycle enzyme activities, elevated ASC and GSH contents, and reduced ROS accumulation, as confirmed by histochemical staining. Furthermore, integrating DAP-seq with transcriptome analysis, we identified 325 direct transcriptional targets of OsNTL2, with a significant enrichment of genes involved in lignin and xylan biosynthesis. Notably, OsNTL2 bound directly to the promoters of, *4-coumarate-CoA ligase 5* (*Os4CL5*), and *cinnamoyl-CoA reductase* (*OsCCR*), activating their transcription. Correspondingly, stress-induced lignin, xylan, and cellulose accumulation was markedly reduced in *ntl2* mutants but enhanced in *OsNTL2*-overexpressing lines. Together, these findings identify OsNTL2 as a key transcriptional regulator that coordinates the ASC-GSH redox cycle and cell wall biosynthesis to confer osmotic stress tolerance in rice.

**Supplementary Information:**

The online version contains supplementary material available at 10.1007/s44154-025-00271-4.

## Introduction

Environmental stresses such as drought, salinity, and extreme temperatures often result in water deficits that cause significant shifts in external water potential, imposing osmotic stress on plants. Osmotic stress is known to induce excessive production of reactive oxygen species (ROS), disrupting cellular redox homeostasis and leading to oxidative damage (Mittler et al. 2022). To mitigate this damage, plants have evolved a sophisticated detoxification system comprising antioxidant enzymes, including ascorbate peroxidase (APX), catalase (CAT), and peroxidase (PRX), alongside non-enzymatic antioxidants such as ascorbate (ASC) and glutathione (GSH) (Foyer and Shigeoka 2011). Central to this defense is the ASC-GSH cycle, a highly coordinated redox network involving APX, monodehydroascorbate reductase (MDHAR), dehydroascorbate reductase (DHAR), and glutathione reductase (GR). This cycle plays a pivotal role in scavenging excess ROS by regenerating ASC and GSH, thereby maintaining redox equilibrium under stress conditions. Dynamic regulation of the ASC-GSH cycle is critical for sustaining cellular redox homeostasis and enabling plants to adapt to diverse abiotic stresses (Angelovici and Mittler 2024; Foyer and Kunert 2024).

Severe abiotic stresses commonly halt plant growth, which fundamentally depends on cell division in meristematic zones followed by extensive cell expansion, a complex process driven by turgor pressure (Schopfer 2006). Consequently, any reduction in cell turgor induced by osmotic stress impairs the cell's ability to expand its polysaccharide network. The plant cell wall plays a crucial role in maintaining turgor pressure, providing structural support, facilitating water transport during growth, and protecting cells from environmental stresses (Tenhaken 2015; Kaur et al. 2024). This dynamic structure is composed primarily of cellulose, non-cellulosic polysaccharides such as hemicellulose and pectin, structural proteins, and, in some cell types, lignin (Zhang et al. 2021). Primary cell walls are heterogeneous and constantly remodeled to respond to environmental and developmental cues, whereas secondary cell walls are more rigid and static, providing mechanical strength to support plant architecture and facilitate water transport (Vaahtera et al. 2019; Colin et al. 2023; Wang et al. 2024a, b).

Alongside cellulose and hemicellulose, lignin constitutes a major component of secondary cell walls (Zhong et al. 2011). Lignin biosynthesis involves a series of enzymes including phenylalanine ammonia-lyase (PAL), cinnamic acid 4-hydroxylase (C4H), ferulate 5-hydroxylase (F5H), 4-coumarate-CoA ligase (4CL), caffeoyl-CoA 3-O-methyltransferase 1 (CCoAOMT), cinnamyl alcohol dehydrogenase (CAD), and cinnamoyl-CoA reductase (CCR) (Yoon et al. 2015). Polymerization of lignin monomers into complex polymers occurs via peroxidases (POD) and laccases (LAC) within the secondary cell wall (Liu et al. 2018). Xylan, a critical hemicellulosic polysaccharide, is essential for assembling distinct cell wall structures. Plant xylans are often extensively O-acetylated, a modification that imparts structural flexibility, enabling xylan to fold and interact with cellulose and lignin (Simmons et al. 2016; Kang et al. 2019). Glycosyltransferase families 43 and 47 (GT43 and GT47) mediate xylan backbone synthesis, while xylan O-acetyltransferases (XOATs) catalyze xylan acetylation (Manabe et al. 2011; Wen et al. 2024).

Previous studies have demonstrated that genes involved in cell wall biosynthesis play critical roles in plant abiotic stress responses (Le Gall et al. 2015). For instance, mutations in cellulose synthase genes such as *CESA6* increase sensitivity to salt stress in Arabidopsis seedling roots (Zhang et al. 2016). Overexpression of *PoCCoAOMT*, encoding caffeoyl-CoA O-methyltransferase, enhances lignin biosynthesis and ROS scavenging, thereby conferring drought tolerance in peony (Zhao et al. 2021). Similarly, *PeLAC10* contributes to drought tolerance in bamboo by participating in lignin polymerization (Li et al. 2020a, b), while overexpression of the laccase-encoding gene *CsLAC4* improves drought resistance in tea plants via enhanced lignin biosynthesis (Yang et al. 2024). Given that salt and drought stresses both alter water potential and induce osmotic stress, these findings underscore the essential role of sustained cell wall biosynthesis in plant adaptation to osmotic challenges. Nevertheless, the transcriptional regulatory mechanisms coordinating cell wall biosynthesis in response to osmotic stress remain largely unknown.

Membrane-bound transcription factors (MTFs) are latent regulators anchored to cellular membranes in an inactive state and become activated upon environmental stimuli. The NAC family (NAM, ATAF1/2, and CUC2) is among the largest plant-specific transcription factor families, characterized by a conserved NAC DNA-binding domain and a flexible transcriptional regulatory domain (TRD) (Nuruzzaman et al. 2013; Xiong et al. 2025). While most NAC proteins localize to the nucleus, a subset termed NAC with Transmembrane Motif 1-like (NTL) proteins harbor a C-terminal transmembrane domain that restricts them to intracellular membranes (Seo et al. 2008). Growing evidence highlights critical roles of NTLs in mediating plant responses to diverse environmental stresses (Shu et al. 2024). In Arabidopsis, NAC016/NTL3 enhances drought tolerance by repressing *Abscisic Acid Responsive Element Binding Protein 1* (*AREB1*) (Sakuraba et al. 2015). *NTL9* expression is induced by osmotic stress and senescence (Yoon et al. 2008), and *NTL6* is upregulated by various abiotic stresses, with truncated *AtNTL6* overexpression conferring enhanced drought resistance (Kim et al. 2012a, b). Similarly, *GmNTL1* is induced by H_2_O_2_ and NaCl, overexpression of GmNTL1 promoted salt tolerance in soybeam (Zhang et al. 2023). In rice, five NTL homologs (OsNTL2-6) have been identified, and OsNTL3 is reported to positively regulate heat tolerance (Liu et al. 2020). However, the transcriptional regulatory network of rice NTLs in osmotic stress responses remains largely unexplored.

In this study, we characterized OsNTL2 as a membrane-associated transcription factor induced by osmotic stress that confers tolerance to osmotic stress in rice. Using DNA affinity purification sequencing (DAP-seq) combined with transcriptome analysis, we identified direct gene targets and delineated the regulatory network of OsNTL2 under osmotic stress conditions. Notably, OsNTL2 directly binds to the promoters of genes encoding key antioxidant enzymes, including *APX2*, *MDHAR1*, *GR2*, *GPX5*, *PRX3/70*, as well as genes involved in cell wall biosynthesis such as *Os4CL5* and *OsCCR*, thereby activating their expression. This coordinated regulation sustains redox homeostasis and promotes stress-induced lignin, xylan, and cellulose accumulation, ultimately enhancing rice tolerance to osmotic stress. Collectively, our results highlight OsNTL2 as a pivotal transcriptional regulator that integrates antioxidant defense and cell wall remodeling to enhance rice osmotic stress resilience, providing valuable molecular targets for crop improvement.

## Results

### The OsNTL2 is a membrane associated transcription factor

**Fig. 1:**
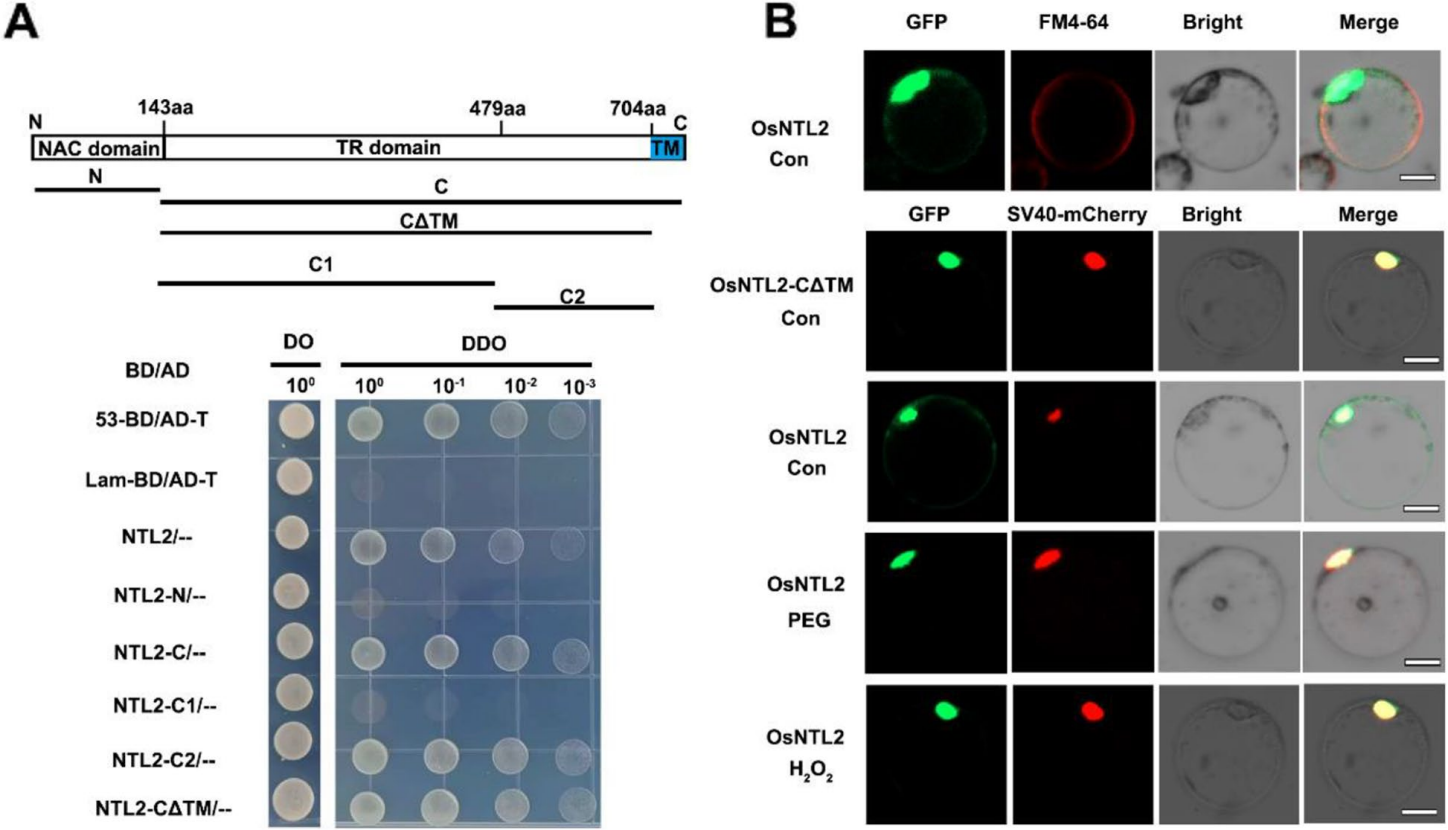
OsNTL2 is a membrane-bound NAC transcription factor. **A** Transactivation activity verification of OsNTL2 in yeast cells. The constructed plasmid pGBKT7-NTL2, pGBKT7-NTL2-N, pGBKT7-NTL2-C, pGBKT7-NTL2-C1, pGBKT7-NTL2-C2, and pGBKT7-NTL2-CΔTM was respectively co-transfected into yeast AH109 with pGADT7. After transformation, all combination of yeasts was grown in SD-Leu/-Trp (DO) and SD-Ade/-His/-Leu/-Trp (DDO) dropout minimal base. **B** Subcellular localization of OsNTL2 in rice protoplasts treated with or without 20% PEG 6000 or H_2_O_2_ for 20 min. FM4-64 was used to highlight plasma membrane, and mCherry was used to show the nucleus. Bar = 10 μm

The TIP subgroup of NTLs transcription factor, including AtNTL6, AtNTL9, and GmNTL1 has been reported that play important role in plant stress response (Ooka et al. 2003; Xiong et al. 2025). In rice, OsNTL2 encodes a 729-amino acid protein belonging to the TIP subgroup and represents its unique member in this species (Fig. S1), although its function remains unclear. Structurally, OsNTL2 features an N-terminal NAC DNA-binding domain and a C-terminal transcriptional activation region (TR) containing a transmembrane (TM) motif (Fig. 1A). To assess its transactivation capacity, we performed a yeast-based GAL4 DNA-binding domain (DBD) reporter assay. Yeast cells expressing full-length OsNTL2 or its C-terminal region exhibited robust growth on synthetic dropout medium lacking leucine, tryptophan, histidine, and adenine, confirming OsNTL2's transactivation activity. In contrast, the N-terminal region lacking the TR failed to support growth, while deletion of the TM motif (residues 704–729, designated CΔTM) did not impair transactivation. Further subdivision of the CΔTM region into C1 (residues 144–479) and C2 (residues 480–704) revealed that the C2 fragment retains transactivation activity. Previous studies have shown that the N-terminal NAC domain mediates homodimer formation, essential for NAC protein function (Xiong et al. 2025). However, yeast two-hybrid assays demonstrated that the N-terminal domain of OsNTL2 does not form homodimers in yeast (Fig. S2).

Subcellular localization analysis was conducted by transiently expressing GFP-tagged OsNTL2 under the *CaMV35S* promoter in rice protoplasts, co-expressed with the nuclear marker mCherry-SV40 or staining with plasma membrane dye, FM4-64. Fluorescence microscopy revealed GFP-OsNTL2 signals overlapping with the nuclear mCherry marker, confirming nuclear localization, alongside plasma membrane-associated GFP signals was also overlapped with fluorescent FM4-64 signals, indicating membrane anchoring of OsNTL2 protein (Fig. 1B). In contrast, the GFP-OsNTL2-CΔTM construct localized exclusively to the nucleus, demonstrating that the TM motif is essential for membrane association (Fig. 1B). Importantly, exposure of rice protoplasts to osmotic stress (20% PEG 6000) or oxidative stress (0.5 mM H_2_O_2_) for 20 min triggered translocation of OsNTL2 from the plasma membrane to the nucleus. Collectively, these results establish OsNTL2 as a membrane-associated transcription factor that relocates to the nucleus in response to osmotic and oxidative stress.

### OsNTL2 positively regulates rice tolerance to salt and osmotic stress

To elucidate the role of OsNTL2 in rice abiotic stress tolerance, we first examined its expression profile under various stress conditions. *OsNTL2* transcripts were modestly upregulated in response to treatments that simulate osmotic stress, including PEG 6000, NaCl, mannitol, and drought (Fig. S3A). A time-course analysis further revealed a gradual increase in *OsNTL2* expression over 48 h of PEG 6000 treatment, confirming its induction by osmotic stress in rice (Fig. S3B).

To investigate the functional significance of OsNTL2 in osmotic stress response, we generated three CRISPR/Cas9-mediated loss-of-function mutants (*ntl2-1/2/3*) and three independent transgenic lines overexpressing *OsNTL2* driven by the *35S* promoter (*OE1/2/3*) (Fig. S4). The mutants harbor frameshift mutations introducing premature stop codons before amino acid 479, predicted to produce truncated, nonfunctional OsNTL2 proteins.

**Fig. 2:**
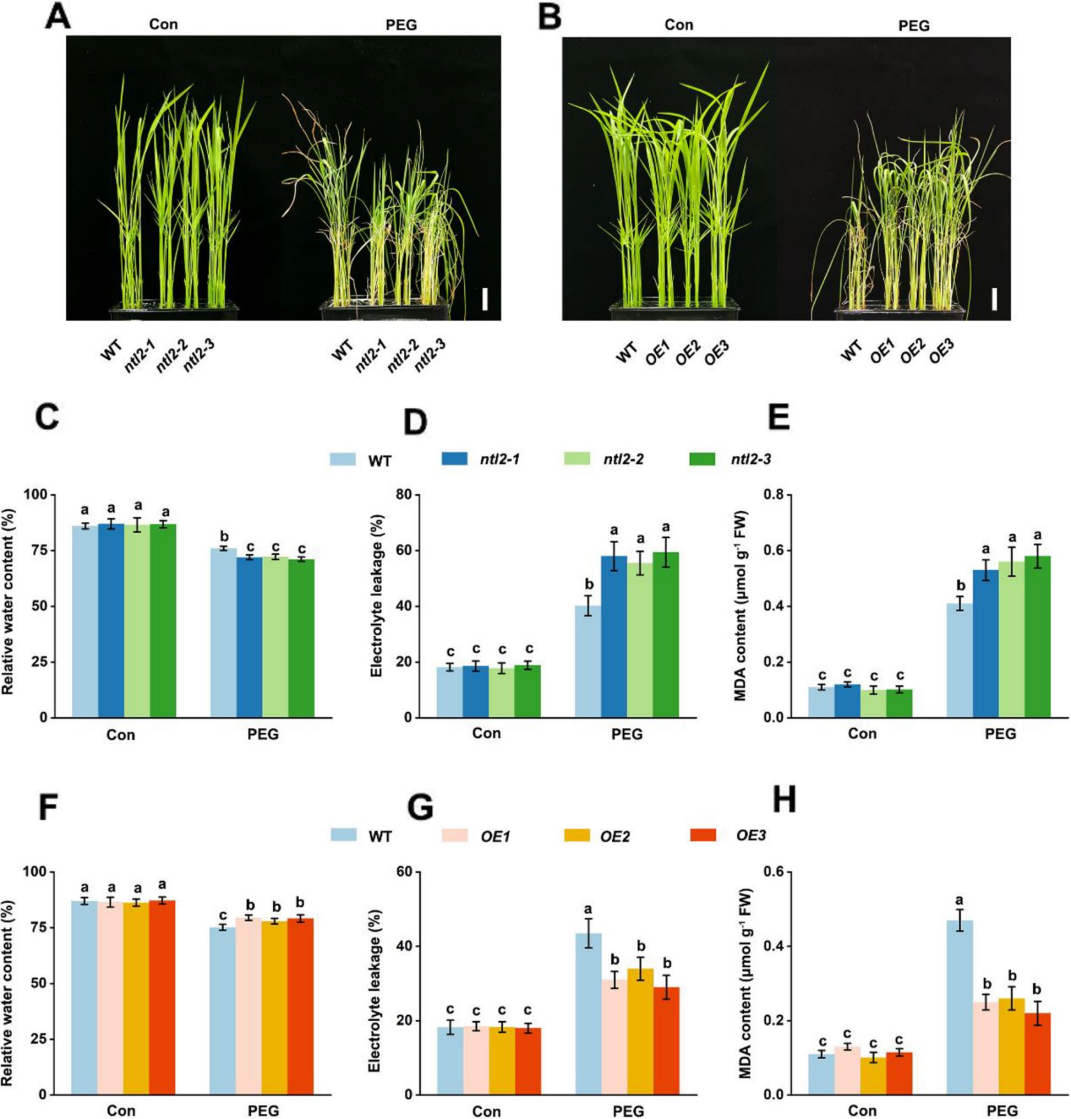
OsNTL2 confers rice osmotic stress tolerance. **A**, **B** Photographs of *ntl2*, *NTL2-OE*, and wild-type (WT) rice seedlings treated starting at 2 weeks after germination with 20% PEG 6000 for 6 days. Scale bars correspond to 2 cm. **C-H** Relative water content (**C**, **F**), malondialdehyde (MDA) content (**E**, **H**) and the percent leakage of electrolytes (**D**, **G**) in the leaves of rice seedlings exposed to PEG 6000 treatment for 4 days. Experiments were repeated at least three times with similar results. Data are presented as mean ± SD (*n* = 3). Different letters denote significant differences at *p* < 0.05 (one-way ANOVA, Duncan’s multiple range test)

Under normal growth conditions, all *ntl2* mutants exhibited no obvious phenotypic differences compared to wild-type (WT) plants (Fig. 2A). However, upon exposure to 20% PEG 6000 or 150 mM NaCl, all *ntl2* mutants showed significantly greater growth inhibition, wilting, chlorosis, and reduced relative water content relative to WT plants (Fig. 2A, C; Fig. S5A). These mutants also exhibited elevated electrolyte leakage (Fig. 2D; Fig. S5B) and increased levels of the oxidative stress marker malondialdehyde (MDA) (Fig. 2E; Fig. S5C), indicating enhanced membrane damage and oxidative stress. In contrast, *OsNTL2*-overexpressing plants displayed improved stress tolerance (Fig. 2B), with reduced growth inhibition, higher relative water content, and lower MDA accumulation and electrolyte leakage following osmotic and salt stress treatments (Fig. 2F-H; Fig. S5). Collectively, these findings demonstrate that OsNTL2 positively regulates rice tolerance to both salt and osmotic stress.

### Genome-wide identification of direct targets of OsNTL2

To elucidate the transcriptional regulatory network mediated by OsNTL2 in rice osmotic stress tolerance, we performed DNA affinity purification sequencing (DAP-seq) to identify its direct binding targets. Recombinant OsNTL2 fused with HaloTag was used to affinity purify sheared genomic DNA from two-week-old wild-type (WT) seedlings, followed by high-throughput sequencing (O'Malley et al. 2016). Two independent biological replicates were prepared, yielding 33,577 and 33,855 enriched peaks respectively, with 8,923 overlapping genes identified as high-confidence OsNTL2 binding targets (Table S1−3).

**Fig. 3:**
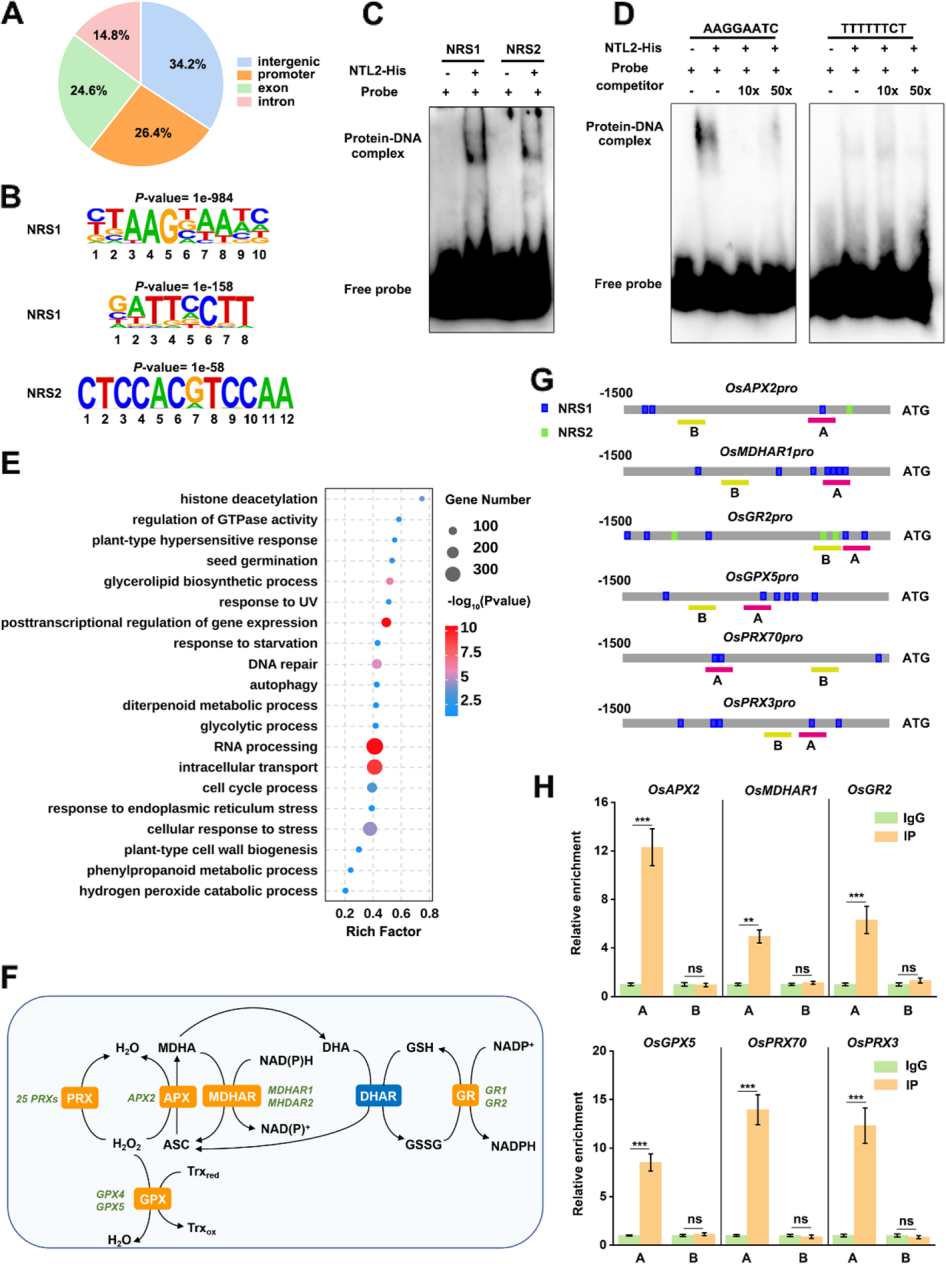
Identification of genome-wide direct targets of OsNTL2. **A** Distribution of OsNTL2 binding sites relative to gene structures. **B** DNA logos of enriched DNA binding sites for OsNTL2 as determined by using HOMER software. **C** Results of EMSAs confirming OsNTL2 bind with NRS1 and NRS2. **D** Specificity of OsNTL2 binding with NRS1 of *OsDREB1C* promoter. The unlabeled probe was taken as competitor. Experiments were repeated three times with similar results. **E** Top enriched GO terms of OsNTL2-bound genes determined by DAP-seq. Bigger dots indicate more genes. **F** Model showed the OsNTL2-bound genes involved in ASC-GSH redox cycle and H_2_O_2_ scavenging. Green font indicated the genes identified in DAP seq results. **G** The promoter structures of the target genes. **H** CUT&Tag-qPCR assays reveal the direct binding of OsNTL2 to the promoters of the indicated genes. The *35S:OsNTL2-eGFP* was transfected into rice protoplasts. After 12 h incubation, protoplasts were used for the CUT&Tag-qPCR assay. A and B indicated the regions used for CUT&Tag-qPCR. The numbers indicate the positions of these motifs relative to the ATG. Values are the percentage of DNA fragments immunoprecipitated with anti-GFP antibodies or IgG relative to the input DNAs. λDNA was used as internal control. Data are presented as mean ± SD (*n* = 3)

Mapping of OsNTL2 binding sites revealed their distribution across various genomic regions: promoter regions (defined as up to 2 kb upstream of transcription start sites) accounted for 26.4%, intergenic regions 34.2%, exons 24.6%, and introns 14.8% (Fig. 3A). To gain insight into OsNTL2 DNA-binding specificity, de novo motif discovery was conducted using Homer software on the DAP-seq enriched regions. This analysis uncovered two significantly enriched motifs, with the top motif represented as (C/T)TAAG(N)AATC or its reverse complement GATTCCTT, designated here as NTL2 Recognition Site 1 (NRS1) (Fig. 3B). Notably, this motif closely resembles the binding site of AtNTL9 (TTAAGTAAT; Weirauch et al. 2014), the Arabidopsis homolog most closely related to OsNTL2, thus validating the DAP-seq findings. A second enriched motif, CTCCACGTCCAA, was identified and termed NTL2 Recognition Site 2 (NRS2).

To experimentally confirm OsNTL2 binding to these motifs, electrophoretic mobility shift assays (EMSA) were performed using purified OsNTL2-His protein and fluorescently labeled DNA probes containing NRS1 or NRS2. Results showed clear binding of OsNTL2 to both motifs (Fig. 3C). As a candidate target gene, *Dehydration-Responsive Element Binding Protein 1C* (*DREB1C*), whose promoter harbors multiple NRS1 sites (Fig. S6A), was examined. EMSA demonstrated specific binding of OsNTL2 to the NRS1-containing promoter probe of *OsDREB1C*, but not to a mutated probe lacking the motif (Fig. 3D). Competition assays with unlabeled probes further confirmed the specificity of this interaction.

To assess the transcriptional activation potential of OsNTL2 in vivo, we generated a reporter construct in which the firefly luciferase gene (LUC) is driven by the *OsDREB1C* promoter (*DREB1Cpro:LUC*), containing multiple NRS motifs. Co-transfection of this reporter with an effector expressing OsNTL2 (eGFP-NTL2) into rice protoplasts resulted in significantly increased LUC activity compared to the empty vector control (eGFP alone), indicating OsNTL2 activates *OsDREB1C* transcription (Fig. S6B). Importantly, treatment with PEG 6000 and H_2_O_2_ further enhanced OsNTL2's transactivation capacity.

Gene ontology (GO) enrichment analysis of OsNTL2 target genes revealed significant overrepresentation of biological processes, including RNA processing, cellular response to stress, intracellular transport, endoplasmic reticulum stress response, and plant-type cell wall biogenesis, indicating that OsNTL2 regulates a broad spectrum of pathways (Fig. 3E). Notably, genes associated with the ascorbate–glutathione (ASC-GSH) cycle and its coupled ROS-scavenging network were enriched. These included key enzymatic components of the cycle such as *cytosolic L-ascorbate peroxidase 2* (*cAPX2*), *monodehydroascorbate reductases* (*OsMDHAR1 and OsMDHAR2*), and *glutathione reductases* (*OsGR1* and *OsGR2*), as well as *glutathione peroxidases* (*GPX4* and *GPX5*) and multiple *peroxidases* (25 members), which participate in the detoxification of H_2_O_2_ associated with ascorbate- glutathione-dependent pathways (Fig. 3F). These results implicate OsNTL2 in the transcriptional regulation of the ASC-GSH cycle and associated antioxidant defense mechanisms.

To validate OsNTL2 binding to the promoters of these ASC-GSH cycle genes in vivo, cleavage under targets and tagmentation (CUT&Tag) followed by qPCR was performed. Promoter regions of *OsAPX2*, *OsMDHAR1*, *OsGR2*, *OsGPX5*, *OsPRX70*, and *OsPRX3* containing NRS1 elements were significantly enriched in OsNTL2 immunoprecipitants compared with IgG controls, confirming the direct association of OsNTL2 with these targets (Fig. 3G, H).

### OsNTL2 regulates the ASC-GSH cycle and maintains H_2_O_2_ homeostasis in rice response to osmotic stress

To investigate the role of OsNTL2 in modulating the ASC-GSH cycle and maintaining H_2_O_2_ homeostasis during rice osmotic stress response, we first analyzed the expression of genes encoding key enzymes involved in the ASC-GSH cycle following PEG 6000 treatment. RT-qPCR results showed that *OsAPX2*, *OsMDHAR1*, *OsGR2*, and *OsGPX5* were upregulated, whereas *OsPRX70* and *OsPRX3* were downregulated in all plants exposed to osmotic stress (Fig. S7). Importantly, the expression levels of these genes were significantly reduced in the *ntl2* mutant but elevated in *OsNTL2* overexpression (*OE*) lines compared with wild-type (WT) plants (Fig. S7), indicating that OsNTL2 positively regulates the transcription of ASC-GSH cycle-related genes under osmotic stress.

**Fig. 4:**
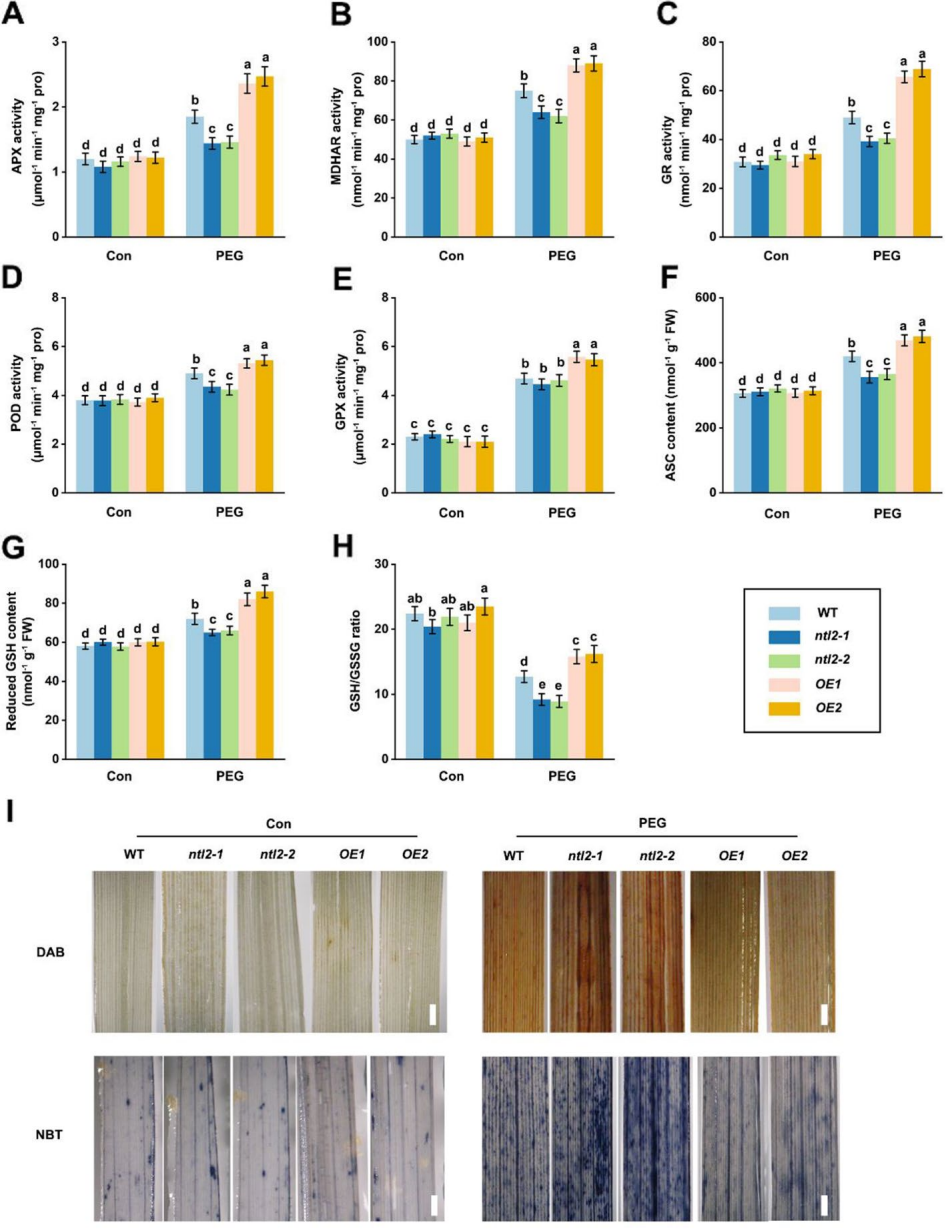
OsNTL2 regulates the AsA-GSH cycle and antioxidant defense to maintain ROS homeostasis in rice under osmotic stress. **A**-**H** Fourteen-day-old *ntl2* mutant, *OsNTL2*-*OE*, and WT plants rice seedlings were subjected to 20% PEG 6000 treatment for 48 h, and the related antioxidant enzyme activities including APX (**A**), MDHAR (**B**), GR (**C**), POD (**D**), and GPX (**E**) and antioxidants, including ascorbate (ASC, **F**), reduced glutathione (GSH, **G**), and GSH/GSSG ratio (**H**) were determined. **I** DAB and NBT staining of leaves from *ntl2* mutant, *OsNTL2*-*OE*, and WT plants treated with or without PEG 6000 for 48 h. Scale bars correspond to 0.2 cm. Different letters denote significant differences at *p* < 0.05 (one-way ANOVA, Duncan’s multiple range test)

We then measured the activities of antioxidant enzymes in WT, *ntl2* mutants, and *OsNTL2-OE* lines under PEG 6000 treatment. The activities of key ASC-GSH cycle enzymes, APX, MDHAR, and GR, as well as peroxidase (POD) were significantly enhanced in WT plants upon stress (Fig. 4A-D). Compared with WT, this stress-induced enzymatic activation was markedly attenuated in *ntl2* mutants but amplified in *OsNTL2-OE* lines. GPX activity showed no significant difference between *ntl2* mutants and WT, whereas higher GPX activity was observed in *OsNTL2-OE* lines (Fig. 4E).

Given the critical roles of APX, MDHAR, and GR in the ASC-GSH cycle, we further quantified ASC and GSH contents in these genotypes. The contents of ASC and GSH followed the same trend as enzyme activities, being decreased in *ntl2* mutants and increased in *OsNTL2-OE* lines relative to WT after PEG 6000 treatment (Fig. 4F-H). Consistent with these biochemical changes, histochemical staining with 3,3-diaminobenzidine (DAB) and nitroblue tetrazolium (NBT) revealed significantly elevated H_2_O_2_ and O_2_^−^ accumulation in *ntl2* mutants and reduced reactive oxygen species levels in *OsNTL2-OE* lines compared to WT under osmotic stress (Fig. 4I). Collectively, these findings demonstrate that OsNTL2 acts as a pivotal positive regulator of the ASC-GSH cycle, thereby contributing to the maintenance of H_2_O_2_ homeostasis and enhancing rice tolerance to osmotic stress.

### Identification of OsNTL2-regulated genes by transcriptome analysis

**Fig. 5:**
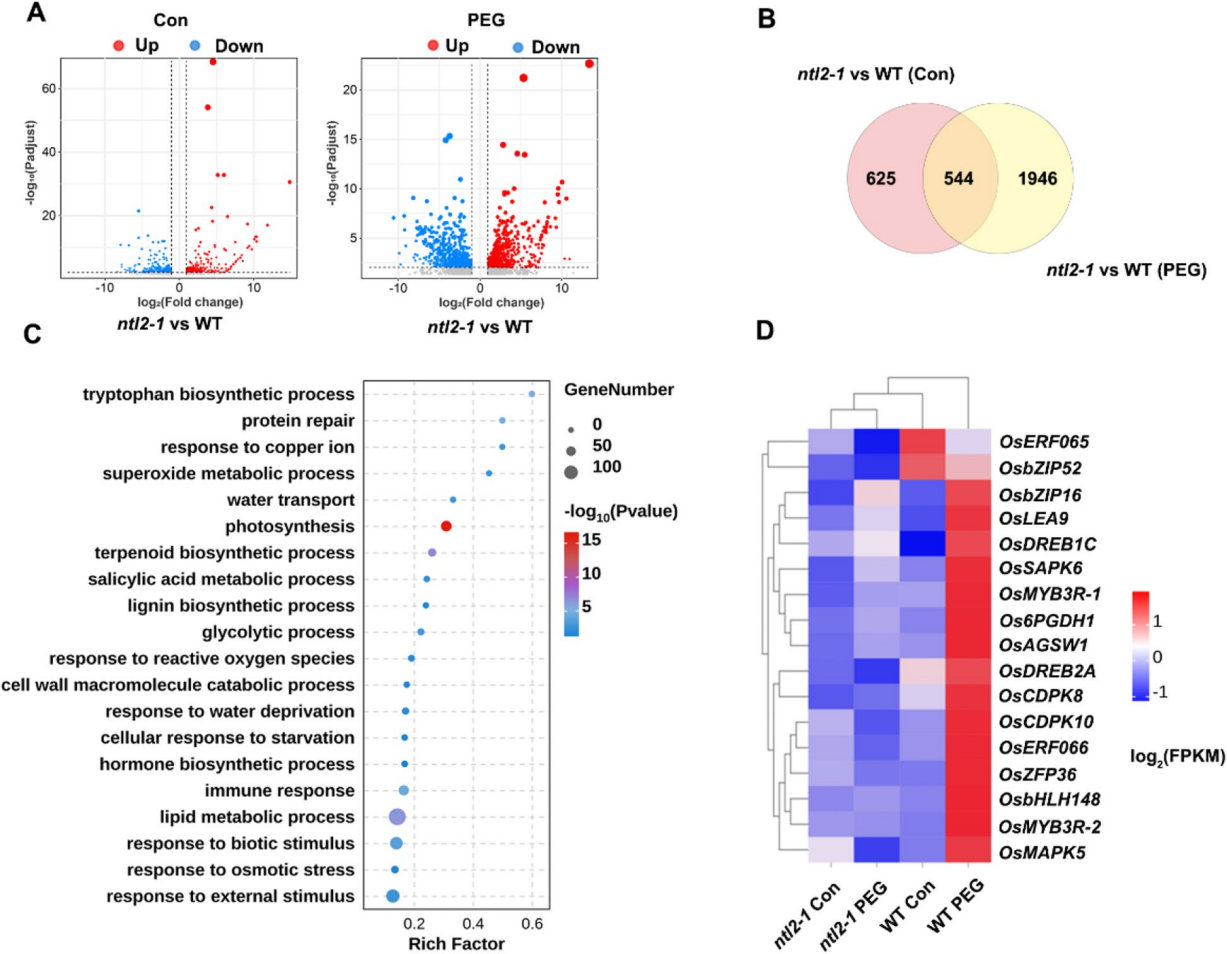
Transcriptome-based identification of *OsNTL2*-regulated genes. **A** The number of the differentially upregulated and downregulated genes in *ntl2* mutant by compared with WT under control and PEG 6000 treatment condition. Fourteen-day-old rice seedlings were subjected to 20% PEG 6000 treatment for 24 h, and gene expression profiles were checked by RNA-Seq analysis. Criteria for differential expression were set as *q* < 0.05, fold change (FC) ≥ 2 for upregulation or FC ≤ 0.5 for down-regulation. **B** Venn diagrams showing the numbers of DEGs regulated by OsNTL2 in both control and PEG 6000 treatment condition. **C** GO analysis of total 3115 OsNTL2-regulated DEGs. **D** Heatmap illustrating the expression patterns of OsNTL2-regulated DEGs associated with water deprivation and osmotic stress response

To identify potential direct transcriptional targets of OsNTL2 in response to osmotic stress, we conducted comparative transcriptome analysis via RNA sequencing (RNA-seq) on two-week-old *ntl2-1* mutant and wild-type (WT) seedlings treated with or without 20% PEG 6000 for 24 h. A total of 3115 OsNTL2-regulated genes were identified as differentially expressed genes (DEGs) between WT and *ntl2-1* seedlings, including 1169 DEGs at control condition, 2490 DEGs at PEG 6000 treatment (*q* < 0.05 and log_2_(fold change) > 1; Fig. 5A, B). Among the 1169 DEGs at control condition, 620 were upregulated and 549 were downregulated in WT (Fig. 5A, Tables S4). Among the 2490 DEGs at PEG 6000 treatment, 1345 were upregulated and 1145 were downregulated in WT (Fig. 5A, Tables S5).

GO enrichment analysis revealed that these OsNTL2-regulated genes were significantly enriched in biological processes including the tricarboxylic acid cycle, photosynthesis, response to osmotic stress, response to water deprivation, and cell wall macromolecule catabolic process, supporting a key role for OsNTL2 in rice osmotic stress adaptation (Fig. 5C). Notably, several well-characterized stress-responsive genes involved in water deprivation responses, such as *OsDREB1C*, *Zinc Finger Protein 36* (*OsZFP36*; Zhang et al. 2014), *Basic Leucine Zipper Transcription Factor 16* (*OsbZIP16*; Chen et al. 2012), *Stress-Activated Protein Kinase 6* (*OsSAPK6*; Jia et al. 2022), and *Mitogen-Activated Protein Kinase 5* (*OsMAPK5*; Xiong and Yang 2003), showed increased expression in WT but not in *ntl2-1* mutants upon PEG 6000 treatment (Fig. 5D).

To validate the transcriptome data, we selected six genes from the aforementioned stress-related biological processes and performed qRT-PCR analysis. The results confirmed that all six genes were significantly upregulated by PEG 6000 treatment in WT seedlings, whereas their induction was markedly attenuated in the *ntl2-1* mutant (Fig. S8). These findings indicate that OsNTL2 confers osmotic stress tolerance in rice by regulating a suite of stress-responsive genes.

### OsNTL2 is involved in the regulation of cell wall biosynthesis in response to osmotic stress

**Fig. 6:**
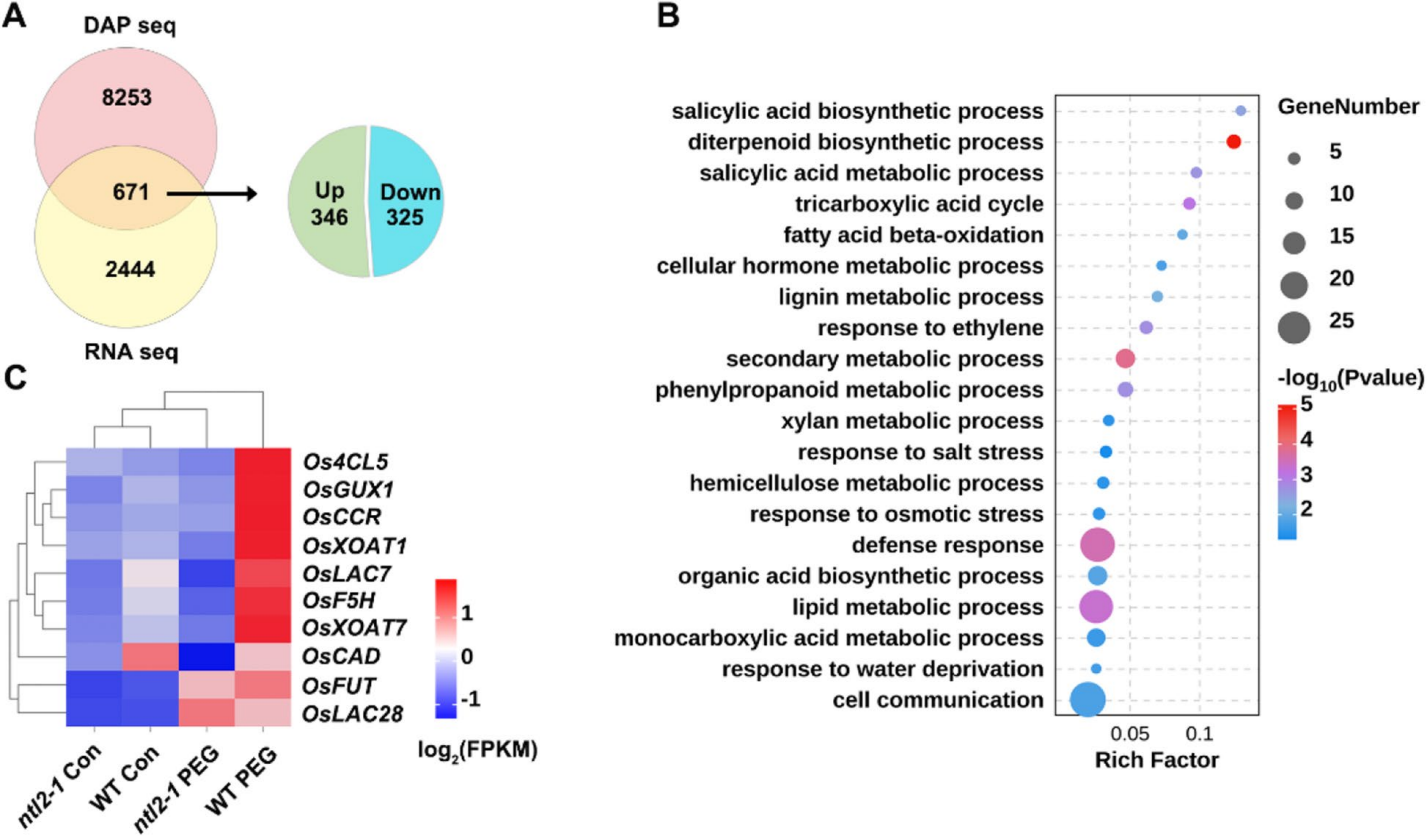
OsNTL2 directly regulates genes involved in cell wall biogenesis. **A** Venn diagram showing the overlap between OsNTL2-bound genes as revealed by DAP-seq and OsNTL2-regulated DEGs identified by RNA-seq. **B** Top enriched Gene Ontology (GO) terms of the 325 direct OsNTL2-regulated target genes identified in (**A**). **C** Heatmap showing the expression of OsNTL2-regulated target genes enriched in lignin and xylan metabolic process as shown in **B**, according to the results from RNA-seq

To identify potential direct target genes of OsNTL2 under osmotic stress, we compared the 8,923 OsNTL2-bound genes identified by DAP-seq with the 3115 OsNTL2-regulated DEGs from RNA-seq. This analysis revealed 671 overlapping genes, representing potential direct targets of OsNTL2, among which 325 were downregulated and 346 were upregulated genes in *ntl2-1* seedlings (Fig. 6A). Considering OsNTL2 function as a transcriptional activator, we choose the 325 downregulated gene as the OsNTL2 specific regulated targets.

GO enrichment analysis of 325 OsNTL2 specific regulated targets showed significant overrepresentation of biological processes such as diterpenoid biosynthetic process, defense response, response to ethylene, and lipid metabolic process, highlighting the diversity of OsNTL2 function (Fig. 6B). Notably, genes related to lignin and xylan metabolic process were also significantly enriched, suggesting that OsNTL2 contributes to the regulation of cell wall biosynthesis under osmotic stress. These included *OsXOAT1* and *OsXOAT7* (*xylan O-acetyltransferases*), *OsGUX1* (*UDP-glucuronate:xylan α-glucuronosyltransferase 1*), *OsFUT* (*xyloglucan fucosyltransferase*) for xylan biosynthesis, and *OsCAD* (*cinnamyl alcohol dehydrogenase*; Takeda et al. 2017), *OsF5H* (*ferulate 5-hydroxylase*), *Os4CL5* (*4-coumarate-CoA ligase 5*), OsCCR (*cinnamoyl-CoA reductase 1*; Li et al. 2020a, b; Borah et al. 2023), *OsLAC7 and OsLAC28* (*laccase*; Liao et al. 2025) for lignin biosynthesis. All of these genes were downregulated in *ntl2-1* compared with WT under PEG 6000 treatment (Fig. 6C).

**Fig. 7:**
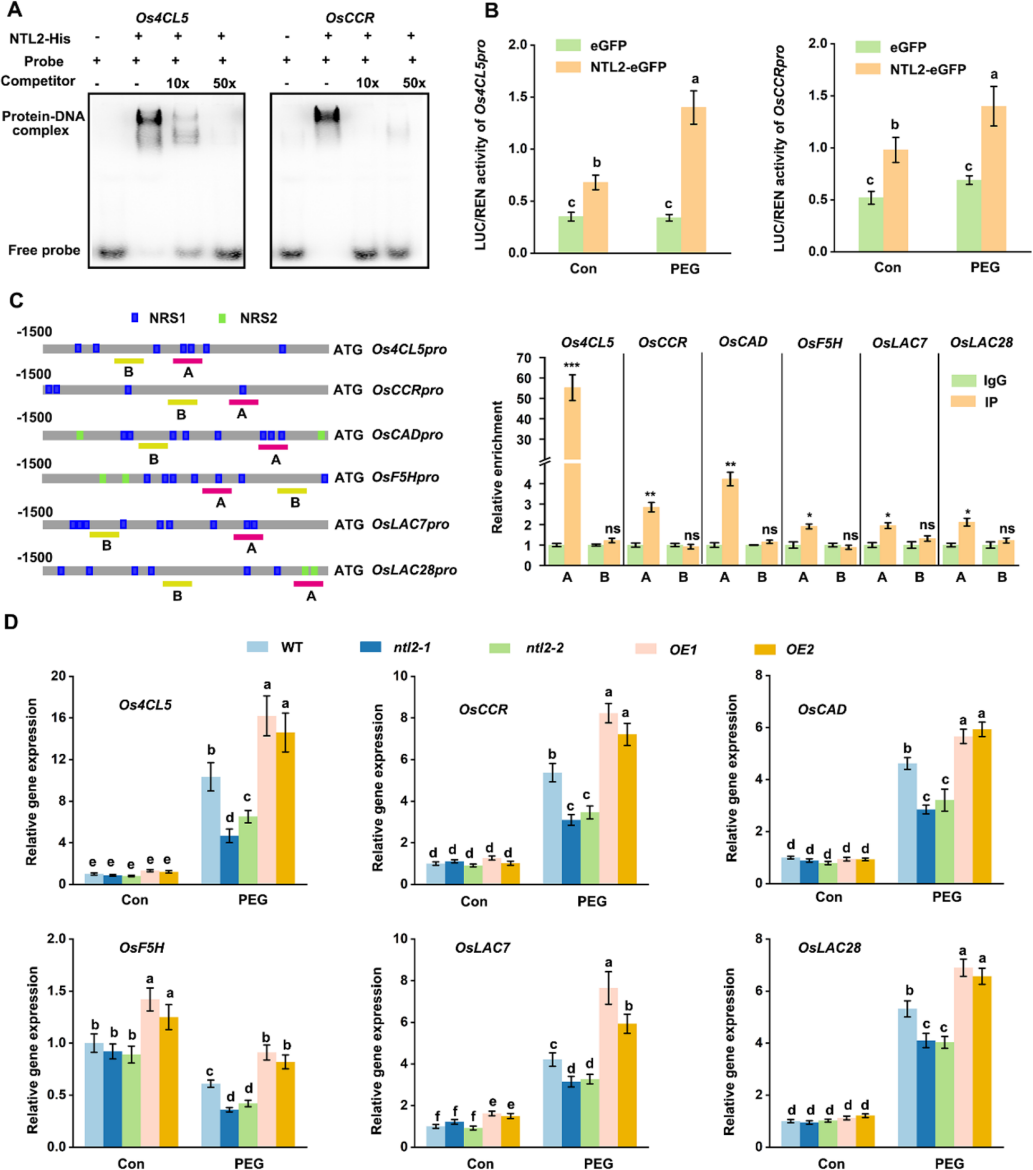
OsNTL2-mediated direct regulation of genes responsible for lignin biosynthesis in rice. **A** EMSA shows that OsNTL2 directly binds to sequence motifs in the*Os4CL5* and *OsCCR* promoters. Recombinant purified OsNTL2 was incubated with biotin-labeled probes or unlabeled DNA probe as competitor. **B** Transient expression assays in rice protoplast showing the transcriptional activities of *Os4CL5* and *OsCCR* were activated by OsNTL2. The upper parts show the effectors and reporters, respectively. *Os4CL5p:LUC* and *OsCCRp:LUC* were co-transformed with empty vector control (eGFP) or OsNTL2-eGFP vectors. After 12 h incubation, protoplasts were treated with 10% PEG 6000 for 30 min. Relative fluorescence signal intensity was then determined. Data are presented as mean ± SD (*n* = 3). Different letters denote significant differences at *p* < 0.05 (one-way ANOVA, Duncan’s multiple range test). **C** CUT&Tag-qPCR assays reveal the direct binding of OsNTL2 to the promoters of the indicated genes. The promoter structures of the target genes are shown (left panel). Blue boxes, OsNTL2 binding motifs as identified by DAP-seq; Green lines, regions used for CUT&Tag-qPCR. The numbers indicate the positions of these motifs relative to the ATG. Values are the percentage of DNA fragments coimmunoprecipitated with anti-GFP antibodies in rice protoplast transiently expressing *OsNTL2-eGFP* or *eGFP* alone relative to the input DNAs (right panel). λDNA was used as internal control. **D** Relative expression of genes involved in lignin biosynthesis in *ntl2* mutant, *OsNTL2*-OE, and WT plants under control and osmotic stress condition. Fourteen-day-old rice seedlings were subjected to 20% PEG 6000 treatment for 24 h, and related gene expression, were determined. Relative gene expression is the gene expression level of plants with treatment divided by that of plants before treatment, both of which were normalized to the expression of *OsACTIN* and *OsUBQ*. Data are presented as mean ± SD (*n* = 3)

We selected *Os4CL5* and *OsCCR* for further validation. EMSA using purified recombinant OsNTL2 confirmed specific binding to promoter fragments of *Os4CL5* and *OsCCR*, as evidenced by shifted bands with biotin-labeled probes containing the identified binding motifs (Fig. 7A). The shifts were abolished by excess unlabeled competitor DNA, confirming binding specificity. Dual-luciferase reporter assays demonstrated that OsNTL2 significantly activated the promoters of *Os4CL5* and *OsCCR* in rice protoplasts compared with an empty vector control, with activation further enhanced under PEG 6000 treatment (Fig. 7B).

All these genes involved in lignin and xylan biosynthesis displayed clear DAP-seq enrichment peaks in their promoter regions (Tables S1 and S2), suggesting direct OsNTL2 binding. Our CUT&Tag-qPCR results further validated that OsNTL2 binds to these promoters in vivo, specifically at the NBS regions (Fig. 7C). Consistently, RT-qPCR analysis revealed that all of these genes were downregulated in *ntl2-1*, but upregulated in *OsNTL2* overexpression lines compared with WT plants under PEG treatment (Fig. 7D and Fig. S9), suggesting that OsNTL2 broadly regulates lignin and xylan biosynthetic pathways under osmotic stress.

**Fig. 8:**
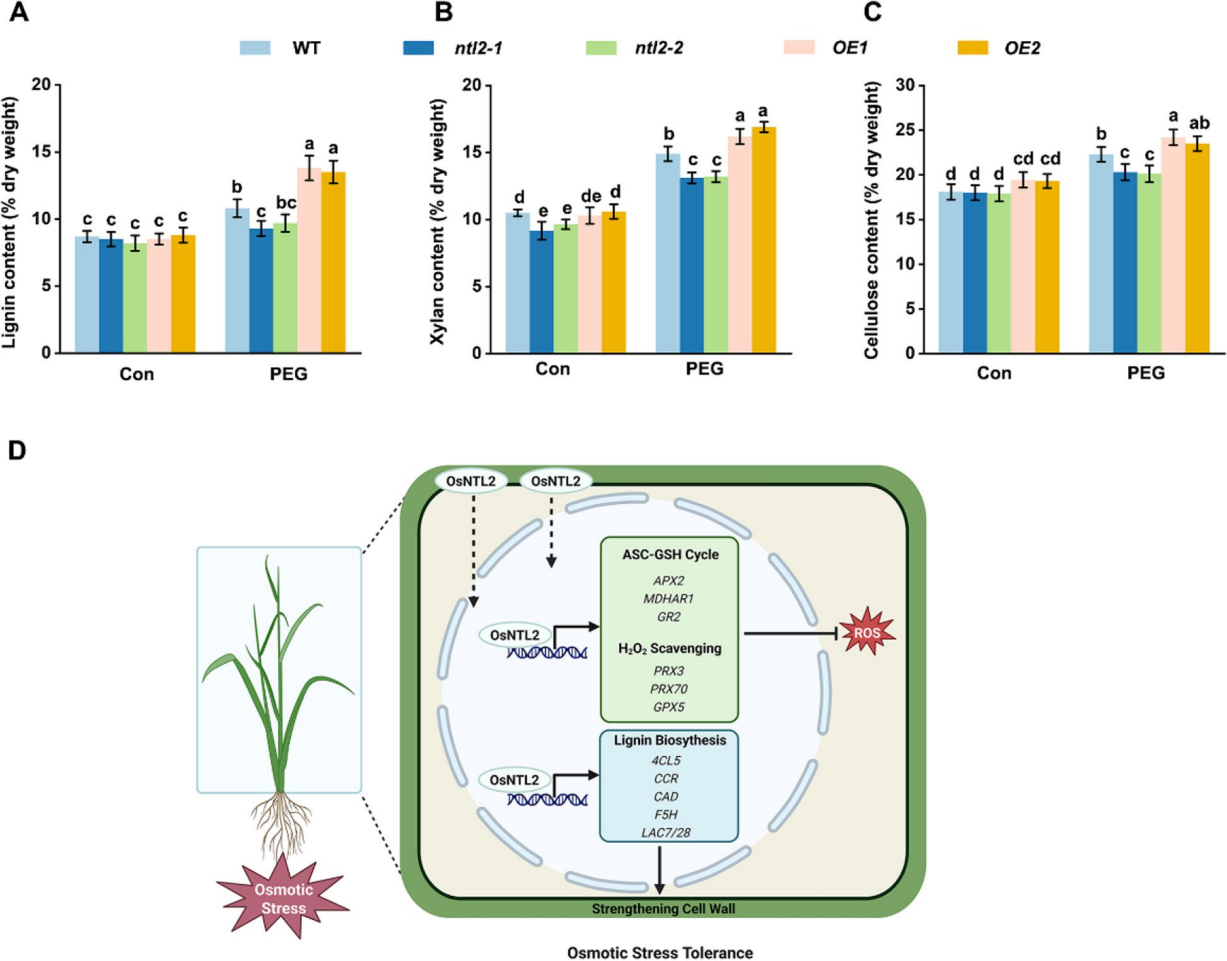
OsNTL2 confers rice osmotic stress by regulating cell wall biosynthesis. **A-C** Lignin, xylan, and cellulose content in *ntl2* mutant, *OsNTL2*-OE, and WT plants under control and osmotic stress condition. Fourteen-day-old rice seedlings were subjected to 20% PEG 6000 treatment for 48 h, and the content of related cell wall components were examined. Different letters denote significant differences at *p* < 0.05 (one-way ANOVA, Duncan’s multiple range test). **D** A proposed molecular model of OsNTL2 modulating ASC-GSH redox cycle and cell wall biosynthesis to confer rice osmotic stress tolerance. Under normal conditions, OsNTL2 localizes to the plasma membrane and nucleus. In response to osmotic stress, OsNTL2 translocate to the nucleus, where OsNTL2 directly activates transcription of the genes encoding ASC-GSH cycle and H_2_O_2_-scavenging enzymes (e.g., *APX2*, *MHDAR1*, *GR2*, *GPX5*, *PRX3*, and *PRX70*) and key enzymes in lignin biosynthesis (e.g.,*Os4CL5*, *OsCCR*, *OsCAD*,*OsF5H*, *OsLAC7*, *and OsLAC28*), strengthening cell wall and finally confers rice osmotic stress tolerance

Cell wall synthesis and remodeling are critical for plant growth and adaptation to abiotic stress. To further validate the role of OsNTL2 in modulating cell wall biosynthesis, we quantified major cell wall components in the *ntl2* mutant and *OsNTL2* overexpression (*OE*) lines. Under normal conditions, cellulose and lignin contents in *ntl2* mutants were comparable to those in WT, whereas xylan content was lower than in WT (Fig. 8A-C). In contrast, *OE* lines exhibited higher cellulose accumulation, but no significant changes in lignin or xylan compared with WT. Upon PEG 6000 treatment, cellulose and lignin contents increased significantly in WT plants. However, stress-induced accumulation of cellulose, xylan, and lignin was attenuated in *ntl2* mutants, but further enhanced in *OE* lines (Fig. 8A-C). These results support that OsNTL2 confers osmotic stress tolerance in rice by activating cell wall biosynthesis.

## Discussion

Water-generated turgor pressure is a major driving force for plant cell expansion. Significant changes in water potential can impose osmotic stress, a physical stimulus that triggers diverse physiological changes at the cellular level, including alterations in turgor pressure, cell wall stiffness and integrity, membrane tension, and cell fluid volume (Yu et al. 2024). Osmotic stress typically leads to a series of morphological, physiological, biochemical, and molecular changes that affect plant growth, development, and productivity (Xiong and Zhu 2002). Consequently, osmotic stress caused by drought and high salinity represents a major environmental constraint on plant growth and agricultural yield (Wang et al. 2024a, b).

Recent studies have highlighted that enhancing antioxidant defense and promoting cell wall biosynthesis are two key strategies by which plants cope with abiotic stresses (Vaahtera et al. 2019; Colin et al. 2023). However, the regulatory mechanisms that coordinate antioxidant defense and cell wall biosynthesis under stress conditions remain poorly understood. Here, we report a central regulatory role of a membrane-associated NAC transcription factor, OsNTL2, in conferring rice osmotic stress tolerance by coordinately regulating antioxidant defense and cell wall biosynthesis.

Most NTL genes are inducible by environmental changes and play key roles in abiotic stress responses (Wang et al. 2016). In Arabidopsis, AtNTL3 enhances drought tolerance by repressing *AREB1* transcription. *atntl3* mutants show improved drought tolerance, while overexpression lines are more sensitive (Sakuraba et al. 2015). Similarly, *AtNTL4* loss-of-function mutants exhibit increased drought resistance by promoting ROS production through direct binding to ROS biosynthesis gene promoters during drought-induced senescence (Lee et al. 2012). In contrast, maize ZmNTLs positively regulate oxidative stress; Arabidopsis plants overexpressing truncated ZmNTL1, ZmNTL2, and ZmNTL5 lacking transmembrane domains are less sensitive to H_2_O_2_ (Wang et al. 2016). These results highlight the diverse and species-specific roles of NTL proteins and their downstream targets in abiotic stress responses.

To function as transcription factors, NTLs must translocate to the nucleus, a process that involves their release, intracellular movement, and nuclear import. NTLs are typically released through proteolytic cleavage, ubiquitin/proteasome-dependent processing, or alternative splicing that omits the transmembrane domain (TMD) (Seo et al. 2008). Evidence indicates that post-translational modifications (PTMs) are essential for the nuclear import of NTLs (Seo et al. 2010). For instance, phosphorylation of NTL6 at Thr142 by SnRK2.8 kinase is critical for its nuclear translocation (Kim et al. 2012a, b), while phosphorylation of plant NTL11 by phosphatidylinositol 4-kinase PI4Kγ5 is necessary for its release and relocalization (Tang et al. 2016). Beyond phosphorylation, other PTMs also play key roles: depalmitoylation of the Medicago truncatula transcription factor MfNACsa is crucial for its nuclear import (Duan et al. 2017). Recently, it was found that oxidative PTM of GmNTL1 at Cys-247 promote its nuclear translocation. Notably, a key regulatory mechanism underlying NTL function involves their stress-triggered translocation from membranes to the nucleus, thereby enabling activation of downstream target genes (Seo et al. 2010). For instance, in *Medicago falcata*, the membrane-associated MfNACsa relocates to the nucleus under drought stress, enhancing drought tolerance through activation of *glyoxalase I* expression and consequent maintenance of the GSH pool (Duan et al. 2017). Similarly, OsNTL3 translocate from the plasma membrane to the nucleus in response to heat stress, and this relocation is essential for heat tolerance in rice via regulation of heat-responsive genes (Liu et al. 2020). More recently, GmNTL1 has been implicated in soybean salt tolerance by directly regulating genes involved in ROS production and Na^+^/K^+^ transport. Notably, salt-induced H_2_O_2_ accumulation promotes GmNTL1 nuclear translocation and transcriptional activity towards its target genes through H_2_O_2_-mediated posttranslational modification on cysteine residues (Zhang et al. 2023).

In our study, we observed that OsNTL2 expression is induced by abiotic stresses and that OsNTL2 positively modulates rice osmotic stress tolerance by activating genes implicated in antioxidant defense and cell wall biosynthesis (Figs. 7, S7, and S9). Under normal conditions, OsNTL2 localizes to both the nucleus and plasma membrane, whereas treatments with PEG 6000 and H_2_O_2_ promote its nuclear accumulation (Fig. 1B). Correspondingly, transcriptional activity of OsNTL2 towards the *OsDREB1C* promoter was significantly elevated in PEG 6000- and H_2_O_2_-treated rice protoplasts compared to controls (Fig. S6). These data suggest that OsNTL2 possibly undergo oxidation in response to osmotic and oxidative stress, which may facilitate its nuclear translocation and function as a transcription factor to regulating downstream gene expression. Given that post-translational modifications (PTMs) are essential for nuclear import of membrane-tethered transcription factors (Zhang et al. 2023), future investigations into the regulatory mechanisms of OsNTL2 translocation under stress will further illuminate the complexity of membrane-tethered TF functional regulation.

Plants have evolved complex regulatory networks for rapid adaptation to environmental changes (Zhang et al. 2021; Leisner et al. 2023; Ravi et al. 2023). Abiotic stress alters gene expression through coordinated *cis*- and *trans*-regulatory elements. Understanding TF binding motifs is key to deciphering gene regulatory networks. Our DAP-seq identified a major OsNTL2 binding motif, NRS1 ((C/T)TAAG(N)AATC), similar to AtNTL9’s recognition sequence (Lindemose et al. 2014), and another motif, NRS2 (CTCCACGTCCAA), containing a CACG element like NAC recognition sites (Puranik et al. 2012). EMSA and CUT&Tag-qPCR confirmed OsNTL2 binds both motifs (Figs. 3C, D, and H), indicating a broad downstream gene network. Such motif-binding versatility parallels observations in strawberry NAC TF FvRIF, a key fruit ripening regulator that binds multiple motifs to activate expression of genes involved in various ripening processes (Li et al. 2023). Given limited knowledge of NTL binding sequences despite conserved DNA-binding domains, our findings offer new candidates for mapping NTL-regulated gene networks.

The ASC-GSH cycle, encompassing enzymes such as APX, MDHAR, DHAR, and GR, constitutes the central antioxidant defense and H_2_O_2_ scavenging system in plants (Foyer and Kunert 2024). APX catalyzes the conversion of H_2_O_2_ to water utilizing ascorbate as the specific electron donor. Additionally, peroxiredoxins (PRXs) participate in H_2_O_2_ elimination through catalytic cycles, playing vital roles in antioxidant defense. Our DAP-seq data revealed that genes involved in ASC-GSH cycle and H_2_O_2_ catabolism are putative OsNTL2 targets (Fig. 3E). Subsequent CUT&Tag-qPCR and RT-qPCR analyses validated direct regulation of *OsAPX2*, *OsMDHAR1*, *OsGR2*, *OsGPX5*, *OsPRX70*, and *OsPRX3* by OsNTL2 (Figs. 3H and S7). Consistently, measurements of antioxidant enzyme activities and histochemical staining for reactive oxygen species demonstrated OsNTL2’s pivotal role in balancing ROS homeostasis during osmotic stress in rice (Fig. 4). In mammals, redox-sensitive TFs such as forkhead box O (FOXO) proteins and nuclear factor erythroid 2-related factor 2 (Nrf2) serve as redox sensors that regulate antioxidant protein levels and transduce stress signals (Priya et al. 2020; Sies et al. 2024). Given the high homology between OsNTL2 and GmNTL1 (Fig. S1), which undergoes H_2_O_2_-mediated cysteine oxidation and nuclear translocation under stress (Zhang et al. 2023), it is plausible that OsNTL2 functions similarly as a redox-sensitive TF central to antioxidant defense during rice stress responses.

Plant cell walls, composed primarily of carbohydrate polymers, lignin, and structural proteins, are essential for maintaining cell shape, mechanical strength, water transport, and turgor pressure resilience. Extensive studies have established close correlations between cell wall metabolism and plant stress responses (Le Gall et al. 2015; Colin et al. 2023). Hemicelluloses, predominantly xylans, contribute substantially to cell wall integrity by interacting with cellulose and lignin, influencing secondary cell wall mechanics and overall plant growth (Kirui et al. 2022). Plant xylans are frequently highly O-acetylated, enabling folding into conformers that bind cellulose microfibrils and lignin. Xylan O-acetyltransferases (XOATs) are critical for maintaining specific acetylation profiles on xylans. Rice mutants deficient in *XOAT6* and *IRX10* exhibit comparable defects in acetyl ester and xylose contents (Wen et al. 2024). We identified *OsXOAT1* and *OsXOAT7*, which are responsible for xylan acetylation and biosynthesis, as direct OsNTL2 targets (Fig. 6). Moreover, expression of *OsXOAT1* and *OsXOAT7* was upregulated in OsNTL2 overexpression lines under PEG 6000 treatment (Figs. S9A and B), consistent with increased xylan content (Fig. 8B), indicating OsNTL2’s regulatory role in xylan formation and cell wall integrity during rice stress responses. Intriguingly, Arabidopsis plants carrying loss-of-function *XOAT1* alleles produce xylan with 50–60% reduced O-acetylation, display collapsed xylem vessels, and exhibit enhanced tolerance to salt, drought, and freezing stresses (Pauly and Ramirez, 2019). However, current evidence suggests that reduced xylan acetylation may not be the direct cause of these phenotypes, leaving the precise role of xylan in plant stress responses unresolved (Pauly and Ramirez 2019). Moreover, stress-induced cellulose accumulation was also significantly reduced in the *ntl2* mutant but enhanced in *OsNTL2* overexpression lines (Fig. 8C), supporting a direct regulatory role of OsNTL2 in cellulose biosynthesis during rice stress response. Increased cellulose synthesis may help maintain cell wall integrity and turgor pressure, facilitating sustained cell growth under water deficit conditions.

The secondary cell wall is reinforced through lignin incorporation. Lignification is a complex process involving multiple phenolic substrates and enzymes (Wang et al. 2013). Lignin reduces water penetration and transpiration through the cell wall, thereby maintaining osmotic balance and membrane integrity (Moura et al. 2010). Enzymes including ferulate 5-hydroxylase (F5H), 4-coumarate CoA ligase (4CL), cinnamyl alcohol dehydrogenase (CAD), and cinnamoyl-CoA reductase (CCR) are key players in monolignols biosynthesis, while laccases that polymerize monolignols to form lignin polymers for cell wall reinforcement (Liao et al. 2025). We found that OsNTL2 binds to promoters of OsCAD, OsF5H, *Os4CL5*,*OsCCR*, OsLAC7, and OsLAC28 and activating their transcription (Fig. 7). Consistently, stress-induced lignin accumulation was markedly diminished in the *ntl2* mutant but enhanced in *OsNTL2* overexpression lines (Fig. 8A), supporting a direct role of OsNTL2 in regulating lignin synthesis during stress responses. Given lignin’s function as a physical barrier against pathogens, these results imply a potential role for OsNTL2 in coordinating plant defense mechanisms. Interestingly, genes involved in defense response and diterpenoid biosynthetic pathways, which constitute major components of plant secondary metabolites critical for defense against pathogens and insects (Cheng et al. 2025), were also identified as direct OsNTL2 targets (Fig. 6B). This suggests that OsNTL2 may act as a master regulator integrating both abiotic and biotic stress responses (Liu et al. 2024). In a similar vein, Arabidopsis NTL9 functions not only as a key regulator of osmotic stress signaling and leaf senescence-associated gene expression but also as an essential player in innate immune responses, including effector-triggered immunity and stomatal defense, through transcriptional regulation of defense- and salicylic acid (SA) biosynthesis-related genes (Yoon et al. 2008; Kim et al. 2012a, b; Block et al. 2014; Zheng et al. 2015; Guo et al. 2017). Future studies aimed at detailed functional dissection and mechanistic understanding of OsNTL2 will be invaluable for elucidating the intricate relationships among cell wall biosynthesis, plant biotic and abiotic stress responses, and overall plant performance.

## Conclusion

Our study reveals that OsNTL2, a membrane-associated NAC transcription factor, enhances rice osmotic stress tolerance by maintaining redox homeostasis and strengthening cell wall biosynthesis. Osmotic stress induces *OsNTL2* expression, and nuclear translocation, thus triggering its binding and activation of genes encoding ASC-GSH cycle and H_2_O_2_-scavenging enzymes (e.g., *APX2*, *MHDAR1*, *GR2*, *GPX5*, *PRX3*, and *PRX70*) and key enzymes in lignin biosynthesis (*Os4CL5*, *OsCCR*, OsCAD, OsF5H, OsLAC7, and OsLAC28). This coordinated regulation boosts antioxidant defense and promotes lignin, xylan, and cellulose synthesis, ultimately improving rice tolerance to osmotic stress. These findings uncover a molecular mechanism linking cell wall architecture modulation with stress adaptation, and identify OsNTL2 as a promising target for engineering or breeding stress-resilient cultivars.

## Materials and methods

### Plant materials and growth condition

Rice (*Oryza sativa* L.) materials used in this study were generated in the background of japonica cultivar “Wuyunjing 7”. *ntl2* mutants were generated with CRISPR/Cas9 according to a previously study (He et al. 2018). The single-guide RNAs (sgRNAs) were designed with CRISPR-P 2.0 (http://crispr.hzau.edu.cn/CRISPR2/) and cloned into the TKC vector. These constructs were transformed into *Agrobacterium tumefacien*s EHA105 for inoculation. Genomic DNA was extracted from the above transgenic plants, and primer pairs flanking the designed target site were used for PCR amplification (Table S8). To obtain transgenic plants overexpressing *OsNTL2*, the full-length open reading frame of *OsNTL2* was inserted into the 1305-GFP vectors, which were provided by the laboratory of Prof. Aying Zhang (College of Life Sciences, Nanjing Agricultural University). After genotyping, homozygous T3 plants were used in this study.

For physiological analysis, seeds were surface-sterilized and germinated in distilled water for 2 days at 30 °C. Germinated seeds were transferred to a growth chamber and grown on 1/2 Murashige and Skoog (1/2 MS) medium. Plants were grown under a 16/8-h (28 °C/25 °C) day/night regime at 150 mmol m^−2^ s^−1^ irradiation for 2 weeks. For analysis of osmotic stress tolerance, 2-week-old seedlings were transferred to 1/2 MS medium with or without 20% PEG 6000 or 150 mM NaCl for 6 days, and then survival rates were calculated after 7 days recovery. The relative water content, MDA content and the percentage of electrolyte leakage were determined from rice seedling leaves harvested four days after the related treatment according to previously reported methods (Li et al. 2024).

### Yeast two hybrid assays

For the transactivation activity verification, the full length and different truncated forms of *OsNTL2* was cloned into the pGBKDT7 vector and then were co-transformed with pGADT7 empty vector into the yeast AH109 strain. For the protein and protein interaction assay, the N terminal of OsNTL2 was separately cloned into pGBKDT7 and pGADT7 vector and co-transformed into the yeast AH109 strain. The yeast cells were grown on -Leu/-Trp or -Leu/-Trp/-His/-Ade medium for 4 days.

### Subcellular localization

Coding sequence of Full-length and CΔTM of *OsNTL2* was fused to GFP in the frame of 1305-GFP vector and GFP-OsNTL2 was transiently expressed in rice protoplasts and its subcellular localization was observed by using confocal microscopy (Zeiss LSM 740). mCherry-SV40 was used to highlight the nucleus and FM4-64 was used to highlight the plasma membrane, respectively.

### Antioxidant enzymes activity

For APX and POD activity, rice seedling leaves were homogenized in 100 mM phosphate buffer solution (pH 7.0). APX activity was determined by monitoring the rate of H_2_O_2_-dependent oxidation of ascorbate (Nakano and Asada 1981; Gambhir et al. 2023). The POD activity, defined as the rate of decomposition of H_2_O_2_, was spectrophotometrically determined with guaiacol as the hydrogen donor at a wavelength of 436 nm (Cai et al. 2008). MDHAR activities were measured according to protocols detailed in Noctor et al. (2016), by monitoring oxidation of NADH or NADPH in the presence of ascorbate and ascorbate oxidase at 340 nm. The activity of GR was measured on the basis of NADPH consumption (Pastori et al. 2000). GPX activity was also monitored by the measurement of NADPH consumption at 340 nm as described by Delaunay et al. (2002).

### Determination of ASC and GSH content

ASC content, total and reduced GSH content was measured according to the previous method (Cui et al. 2011).

### Histochemical staining of H_2_O_2_ and O_2_^−^

For DAB staining,10 mg DAB powder and 0.05% Tween-20 was dissolved in 10 mL ddH_2_O (pH 3.8; Li et al. 2025). The rice leaves were taken and submerged into DAB solution and incubated at room temperature overnight under dark condition. For NBT staining, rice leaves were submerged into NBT solution in 10 mM potassium phosphate buffer (pH 7.8). Then the stained leaves were washed with 75% ethanol until the chlorophyll was completely removed and photographed (model Stemi 2000-C; Carl Zeiss, Germany).

### DAP-seq and data analysis

DAP-seq was conducted according to the procedure previously reported (Bartlett et al. 2017). In brief, genomic DNA from the leaves of 14-day-old seedlings. Fragmented gDNA were constructed into libraries using the NEXTFLEX Rapid DNA Seq Kit (PerkinElmer, Inc., Austin, TX, USA). Recombinant OsNTL2 was produced as a fusion protein with the HaloTag using the TNT SP6 Coupled Wheat Germ Extract System (Promega, Madison, WI, USA). Magne Halo Tag Beads (Promega) were utilized to purify and capture the expressed protein. The OsNTL2-bound beads were incubated with adapter ligated gDNA libraries. Eluted DNA, were sequenced on Illumina NavoSeq6000 with two biological duplicates. Without the addition of protein to beads were taken as the input negative control DAP libraries. DAP-seq reads were aligned to the reference genome (MSU) using Bowtie2 (Langmead and Salzberg 2012). MACS2 callpeak (Zhang et al. 2008) and IDR software were used to merge the peaks of the two biological duplicates with *P* < 0.05, and to score the reliability of these repeated peaks. The OsNTL2 binding regions included promoter (up to 2-kb upstream from the TSS), intergenic region, intron, exon, 3′ UTR, and 5′UTR. Motifs were discovered using Homer version 3 (Heinz et al. 2010). GO enrichment analysis was performed based on the Gene Ontology Resource database (http://geneontology.org/).

### RNA-seq and data analysis

14-day-old rice seedlings were treated with or with 20% PEG 6000 for 24 h, and leaves were harvested for total RNAs extraction. RNA-seq were performed at Novogene (Beijing, China). Total RNA was used as input material for the RNA sample preparations. Briefly, mRNA was purified from total RNA. Fragmentation was carried out using divalent cations under elevated temperature in First Strand Synthesis Reaction Buffer(5X). First strand cDNA was synthesized using random hexamer primer and M-MuLV Reverse Transcriptase (RNase H-). Second strand cDNA synthesis was subsequently performed using DNA Polymerase I and RNase H. Remaining overhangs were converted into blunt ends via exonuclease/polymerase activities. After adenylation of 3' ends of DNA fragments, Adaptor with hairpin loop structure were ligated to prepare for hybridization. Then the library fragments were purified with AMPure XP system (Beckman Coulter, Beverly, USA) and amplified by PCR with universal PCR primers and index (X) primer. The clustering of the index-coded samples was performed on a cBot Cluster Generation.

System using TruSeq PE Cluster Kit v3-cBot-HS (Illumia) according to the manufacturer's instructions. After cluster generation, the library preparations were sequenced on an Illumina Novaseq platform and 150 bp paired-end reads were generated. Hisat2 v2.0.5 was used to analyze the RNA-seq clean reads reference the rice genome (the rice annotation project, RAP-DB). Genes with *P* < 0.05 and log2_ratio over 1 were identified as DEGs using DEseq2. GO analysis of these DEGs were conducted by using OmicShare online software.

#### EMSA

The EMSA assay was performed using an EMSA Probe Biotin Labeling Kit and a Chemiluminescent EMSA Kit (Beyotime) according to the protocol. The labeled probes (0.5 mM) were incubated with purified proteins (2 mg fusion protein per reaction) in 10 μL mixtures at room temperature for 20 min. To determine the specificity of the DNA–protein binding reactions, competition experiments were performed with 50- and 200-fold excess unlabeled probes. Labeled probes and the shifted DNA–protein complexes were visualized using a chemiluminescence apparatus (Tanon 5200 Multi, Tanon Biomart).

### Dual luciferase assay

Promoter fragments of *OsDREB1C*, *OsCESA3*, *OsCCR*, and *Os4CL5* were cloned into the pGreenII0800:Luc vector and then co-transformed with *35S:eGFP-OsNTL2* or *35S:eGFP* vector into the rice protoplasts. After 12 h incubation, protoplasts were treated with or without 10% PEG 6000, or 1 mM H_2_O_2_ for 30 min. The luciferase activity was measured after cell lysis using the Dual Luciferase Reporter Gene Assay Kit (Beyotime). The related ratio of firefly to Renilla LUC fluorescence intensity was quantified.

### Cell wall compositions determination

Cellulose content assay was carried out according to the protocols described in the Solarbio kit (BC4280). lignin content assay was carried out according to the protocols described in the Solarbio kit (BC4200). Xylan content assay was carried out according to the protocols described in the Solarbio kit (BC4394). The experiments were repeated at least three times.

### CUT&Tag-qPCR

DNA was prepared from rice protoplast expressing *35S:eGFP-OsNTL2* or *35S:eGFP* by using CUT-Tag kit (Novoprotein, SuZhou); three biological replicates were included in the assay. For the PCR reaction, the promoter of Actin2 was used as a reference, and DNA amplification data for the CUT&Tag samples were normalized to the input samples. The primers used to amplify the enriched regions of the target genes are listed in Table S8.

### RT-qPCR

Total RNA was isolated from the leaves of rice seedlings after different treatments at indicated time points using the TRIzol reagent according to the manufacturer's instructions. qPCR was performed using a Mastercycler ep realplex Real-time PCR System (Eppendorf, Hamburg, Germany) in a reaction mixture of 20 ml with SYBR Premix Ex Taq (TaKaRa Bio, China) according to the manufacturer's instructions. Primers and for the corresponding genes are listed in Table S8. Relative expression levels of corresponding genes are presented relative to values of the corresponding control samples at the indicated times or under the indicated conditions after normalization to transcript levels of *OsACTIN* and *OsUBQ*.

### Statistical analysis

All data were analyzed using SPSS 23.0 (SPSS, Chicago, IL, USA). Comparisons were performed by independent sample *t*-test (two-tailed) or one-way ANOVA based on Duncan's multiple range test (two-tailed).

## Supplementary Information

Supplementary Material 1: Figure S1. Phylogenetic analysis of NTLs protein from Arabidopsis, rice, and soybean. The amino acid sequences of the NAC domains of NTLs were analyzed using the BioEdit software (http://www.mbio.ncsu.edu/). Subfamily names were annotated according to Ooka et al.2003 with each family with different colors. Figure S2. OsNTL2 does not forms a homodimer through its N terminal. Figure S3. Gene expression of OsNTL2 was induced by abiotic stresses. A Two-week-old rice seedlings were treated with 20% PEG 6000, 150 mM NaCl, 300 mM Mannitol and dehydration for 12 h, respectively. And then leave sample were taken. The expression level of OsNTL2 was examined by qPCR. B Expression of *OsNTL2* in rice seedlings under PEG 6000 treatment. Relative gene expression is the gene expression level of plants with treatment divided by that of plants before treatment, both of which were normalized to the expression of OsACTIN and OsUBQ. Figure S4. The characterization of *ntl2* mutants and *OsNTL2*-overexpression lines. A Loss-of-function mutants of OsNTL2 generated by CRISPR/Cas9-based genome editing which were named as *ntl2-1*, *ntl2-2*, and *ntl2-3*, respectively. B Relative expression level of *OsNTL2* in three OE lines were determined. Relative gene expression is the gene expression level of OE plants divided by that of WT plants, both of which were normalized to the expression of OsACTIN and OsUBQ. Figure S5. OsNTL2 confers rice salt stress tolerance. A Photographs of* ntl2*, *NTL2-OE*, and wild-type (WT) rice seedlings treated starting at 2 weeks after germination with 150 mM NaCl for 6 days. Scale bars correspond to 2 cm. B-C Malondialdehyde (MDA) content and the percent leakage of electrolytes in the leaves of rice seedlings exposed to NaCl treatment for 4 days. experiments were repeated at least three times with similar results. Data are presented as mean ± SD (*n* = 3). Different letters denote significant differences at *p* < 0.05 (one-way ANOVA, Duncan’s multiple range test). Figure S6. Dual luciferase assay to confirm the transactivation activity of OsNTL2. A Schematic diagram indicated the NRS sites on the promoter of OsDREB1C. B The *35S:eGFP-OsNTL2* effector or the empty vector (eGFP) was co-transfected with *OsDREB1Cpro:LUC* reporter constructs into rice protoplasts. After 12 h incubation, protoplasts were treated with 20% PEG 6000, or 0.5 mM H_2_O_2_ for 30 min. Relative fluorescence signal intensity was then determined. Data are presented as mean ± SD (*n* = 3). Different letters denote significant differences at *p* < 0.05 (one-way ANOVA, Duncan’s multiple range test). Figure S7. OsNTL2 regulates the expression of gene encoding antioxidant enzymes in response to osmotic stress. *OsACTIN* and *OsUBQ* was used as an internal control. Data are presented as mean ± SD (*n* = 3). Different letters denote significant differences at *p* < 0.05 (one-way ANOVA, Duncan’s multiple range test). Figure S8. Verification of gene expression by RT-qPCR. *OsACTIN* and *OsUBQ* was used as an internal control. Asterisks indicate significant differences (**P < 0.01, ***P < 0.001; Student’s *t*-test). Figure S9. Relative expression of genes involved in xylan biosynthesis in *ntl2* mutant, *OsNTL2-OE*, and WT plants under control and osmotic stress condition. Fourteen-day-old rice seedlings were subjected to 20% PEG 6000 treatment for 24 h, and related gene expression were determined. Relative gene expression is the gene expression level of plants with treatment divided by that of plants before treatment, both of which were normalized to the expression of *OsACTIN* and *OsUBQ*. Data are presented as mean ± SD (*n* = 3).

Supplementary Material 2: Table S1. List of DAP-seq enriched peaks in biological replicate 1. Table S2. List of DAP-seq enriched peaks in biological replicate 2. Table S3. 8923 mapping targets of OsNTL2 from DAP seq results. Table S4. List of differentially expressed genes in ntl2 mutant compared with WT under control condition. Table S5. List of differentially expressed genes in ntl2 mutant compared with WT under PEG treatment. Table S6 671 common genes identified in both DAP seq and RNA seq. Table S7 325 OsNTL2 specific regulated targets. Table S8. Primers used in this study.

## Data Availability

The data supporting the findings of this study are available within the article and its supplementary materials, and also are available from the corresponding author upon reasonable request.

## Abbreviations

APX: Ascorbate peroxidase
ASC: Ascorbate
CAD: Cinnamyl alcohol dehydrogenase
CCR: Cinnamoyl-CoA reductase
CESAs: Cellulose synthases
CUT&Tag: Cleavage under targets and tagmentation
DAB: 3,3′-Diaminobezidine
DAP-seq: DNA affinity purification-sequencing
DEGs: Differentially expressed genes
F5H: Ferulate 5-hydroxylase
GPX: Glutathione peroxidase
GR: Glutathione reductase
GSH: Glutathione
LAC: Laccase
MDA: Malondialdehyde
MDHAR: Monodehydroascorbate reductase
NTL: NAC with transmembrane motif1-like
PEG: Polyethylene glycol
PRX: Peroxidase
ROS: Reactive oxygen species
RT-qPCR: Reverse transcription-quantitative polymerase chain reaction
SCW: Secondary cell wall
TM: Transmembrane
XOAT: Xylan O-acetyltransferase
