# Supplementary material for: OsNTL2 confers rice osmotic stress resilience through coordinated transcriptional regulation of the ASC-GSH redox cycle and cell wall biosynthesis
Source: Stress Biol. 2025 Nov 27;5(1):72. doi: 10.1007/s44154-025-00271-4 (PMC12657681; doi:10.1007/s44154-025-00271-4)
Supplement: Supplementary file 1 — Supplementary Material 1: Figure S1. Phylogenetic analysis of NTLs protein from Arabidopsis, rice, and soybean. The amino acid sequences of the NAC domains of NTLs were analyzed using the BioEdit software (http://www.mbio.ncsu.edu/). Subfamily names were annotated according to Ooka et al.2003 with each family with different colors. Figure S2. OsNTL2 does not forms a homodimer through its N terminal. Figure S3. Gene expression of OsNTL2 was induced by abiotic stresses. A Two-week-old rice seedlings were treated with 20% PEG 6000, 150 mM NaCl, 300 mM Mannitol and dehydration for 12 h, respectively. And then leave sample were taken. The expression level of OsNTL2 was examined by qPCR. B Expression of OsNTL2 in rice seedlings under PEG 6000 treatment. Relative gene expression is the gene expression level of plants with treatment divided by that of plants before treatment, both of which were normalized to the expression of OsACTIN and OsUBQ. Figure S4. The characterization of ntl2 mutants and OsNTL2-overexpression lines. A Loss-of-function mutants of OsNTL2 generated by CRISPR/Cas9-based genome editing which were named as ntl2-1, ntl2-2, and ntl2-3, respectively. B Relative expression level of OsNTL2 in three OE lines were determined. Relative gene expression is the gene expression level of OE plants divided by that of WT plants, both of which were normalized to the expression of OsACTIN and OsUBQ. Figure S5. OsNTL2 confers rice salt stress tolerance. A Photographs of ntl2, NTL2-OE, and wild-type (WT) rice seedlings treated starting at 2 weeks after germination with 150 mM NaCl for 6 days. Scale bars correspond to 2 cm. B-C Malondialdehyde (MDA) content and the percent leakage of electrolytes in the leaves of rice seedlings exposed to NaCl treatment for 4 days. experiments were repeated at least three times with similar results. Data are presented as mean ± SD (n = 3). Different letters denote significant differences at p < 0.05 (one-way ANOVA, Duncan’s multiple range te [file 44154_2025_271_MOESM1_ESM.docx]

**Supporting information**

**Figure S1**

**
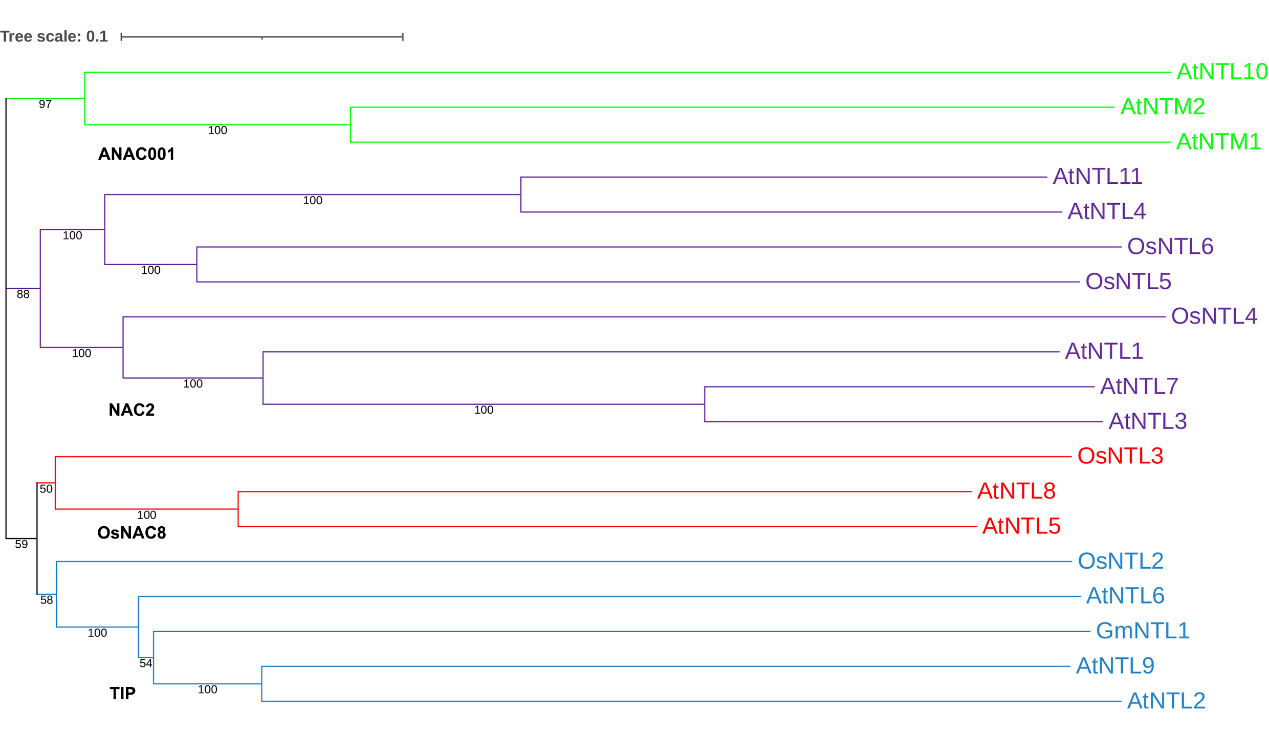
**

**Figure S1. Phylogenetic analysis of NTLs protein from Arabidopsis, rice, and soybean.** The amino acid sequences of the NAC domains of NTLs were analyzed using the BioEdit software (http://www.mbio.ncsu.edu/). Subfamily names were annotated according to Ooka et al.2003 with each family with different colors.

**
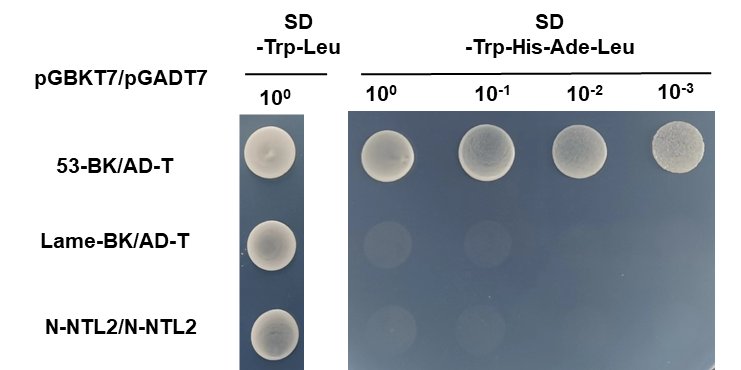
Figure S2**

**Figure S2. OsNTL2 does not forms a homodimer through its N terminal.**

**Figure S3**

**
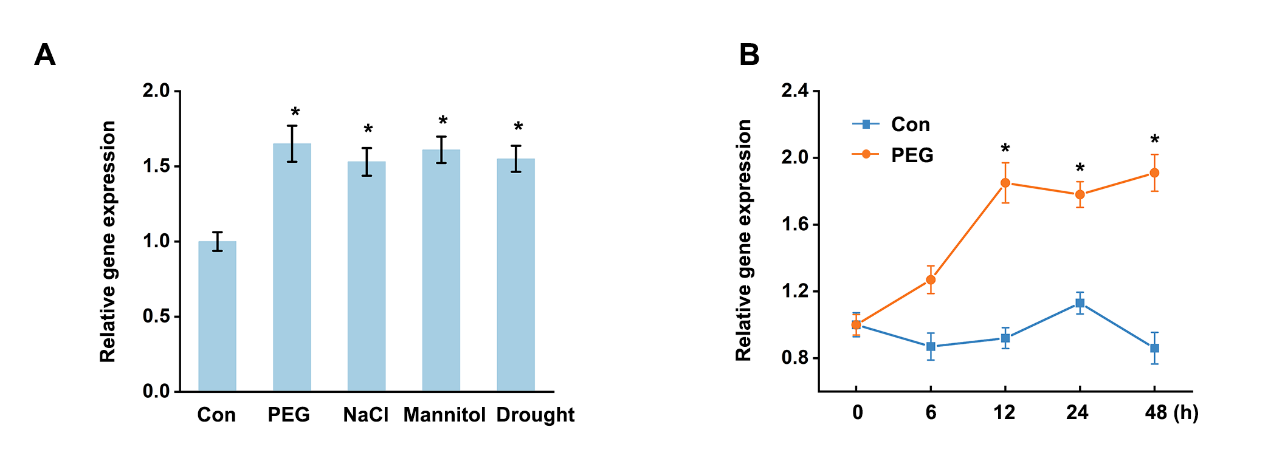
Figure S3. Gene expression of *OsNTL2* was induced by abiotic stresses.** **A** Two-week-old rice seedlings were treated with 20% PEG 6000, 150 mM NaCl, 300 mM Mannitol and dehydration for 12 h, respectively. And then leave sample were taken. The expression level of *OsNTL2* was examined by qPCR. **B** Expression of *OsNTL2* in rice seedlings under PEG 6000 treatment. Relative gene expression is the gene expression level of plants with treatment divided by that of plants before treatment, both of which were normalized to the expression of *OsACTIN* and *OsUBQ*.

**Figure S4**

**
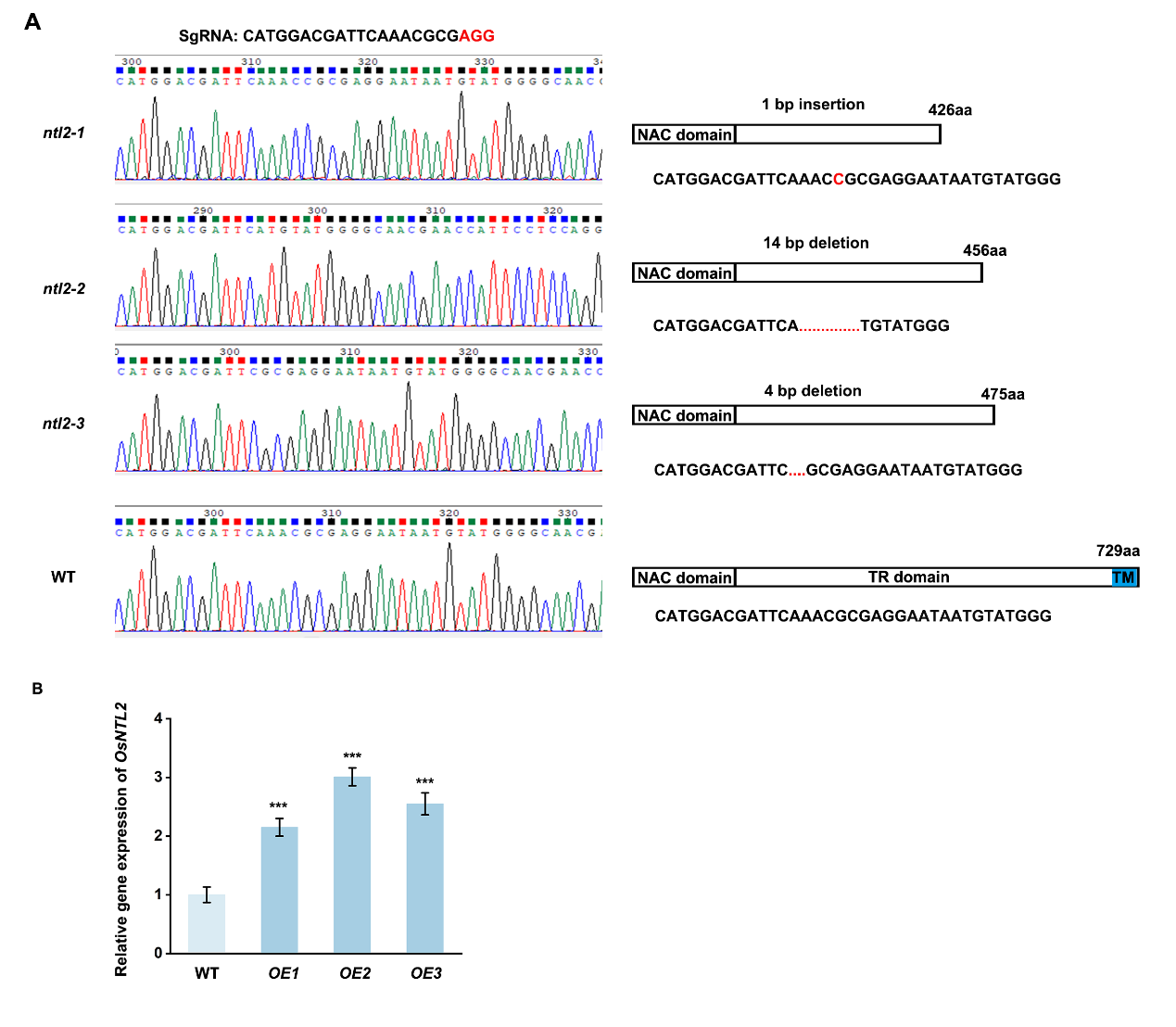
**

**Figure S4. The characterization of *ntl2* mutants and *OsNTL2*-overexpression lines.** A Loss-of-function mutants of OsNTL2 generated by CRISPR/Cas9-based genome editing which were named as *ntl2-1*, *ntl2-2*, and *ntl2-3*, respectively. B Relative expression level of *OsNTL2* in three OE lines were determined. Relative gene expression is the gene expression level of *OE* plants divided by that of WT plants, both of which were normalized to the expression of *OsACTIN* and *OsUBQ*.

**Figure S5**

**
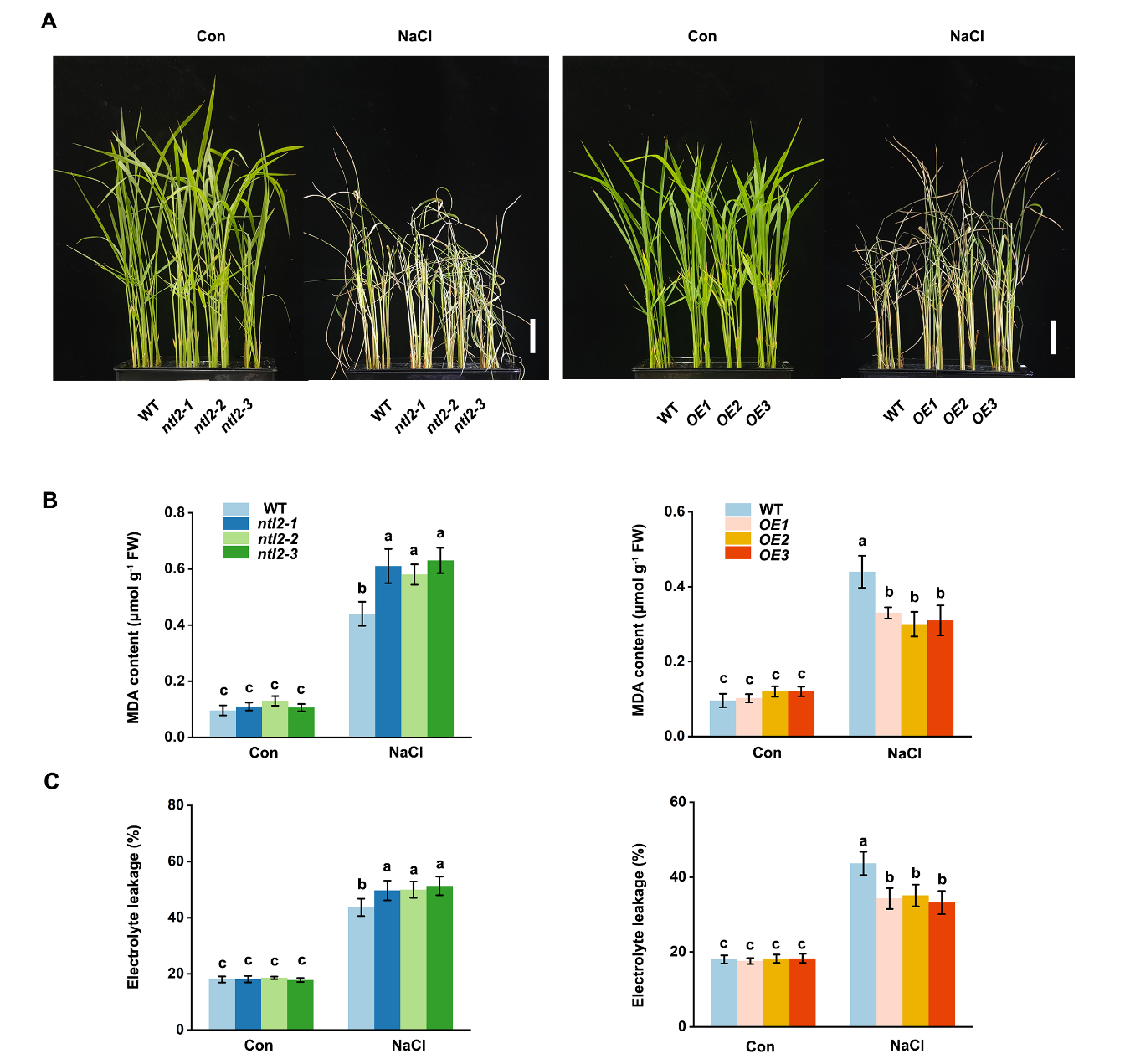
**

**Figure S5. OsNTL2 confers rice salt stress tolerance.** **A** Photographs of *ntl2*, *NTL2-OE*, and wild-type (WT) rice seedlings treated starting at 2 weeks after germination with 150 mM NaCl for 6 days. Scale bars correspond to 2 cm. **B-C** Malondialdehyde (MDA) content and the percent leakage of electrolytes in the leaves of rice seedlings exposed to NaCl treatment for 4 days. experiments were repeated at least three times with similar results. Data are presented as mean ± SD (n = 3). Different letters denote significant differences at *p* < 0.05 (one-way ANOVA, Duncan’s multiple range test).

**Figure S6**

**
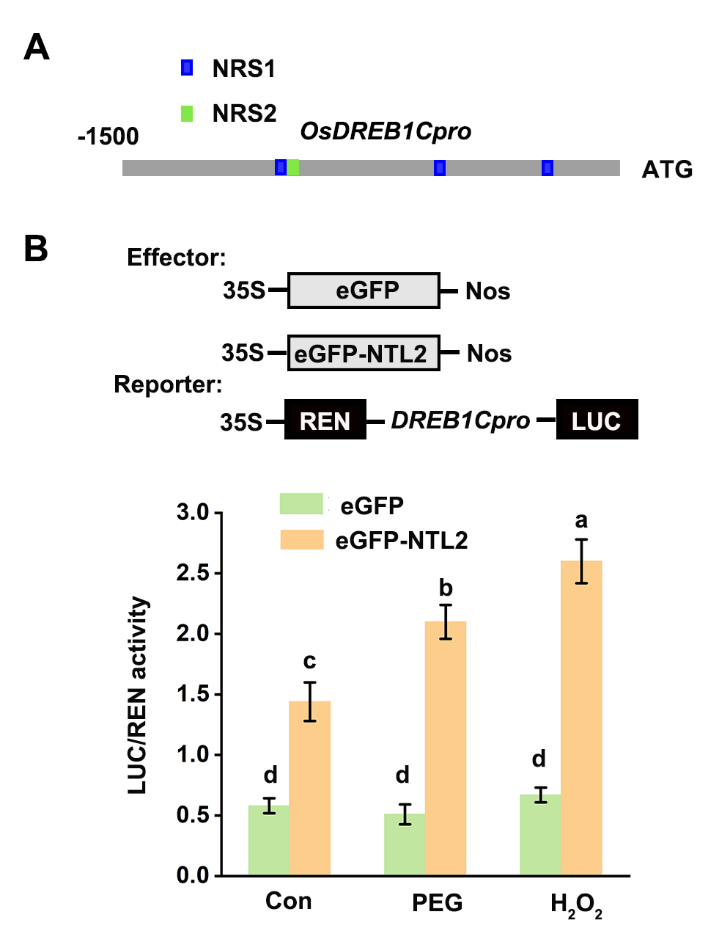
**

**Figure S6. Dual luciferase assay to confirm the transactivation activity of OsNTL2**. **A** Schematic diagram indicated the NRS sites on the promoter of *OsDREB1C*. **B** The *35S: eGFP-OsNTL2* effector or the empty vector (eGFP) was co-transfected with *OsDREB1Cpro:LUC* reporter constructs into rice protoplasts. After 12 h incubation, protoplasts were treated with 20% PEG 6000, or 0.5 mM H_2_O_2_ for 30 min. Relative fluorescence signal intensity was then determined. Data are presented as mean ± SD (n = 3). Different letters denote significant differences at *p* < 0.05 (one-way ANOVA, Duncan’s multiple range test).

**Figure S7**

**
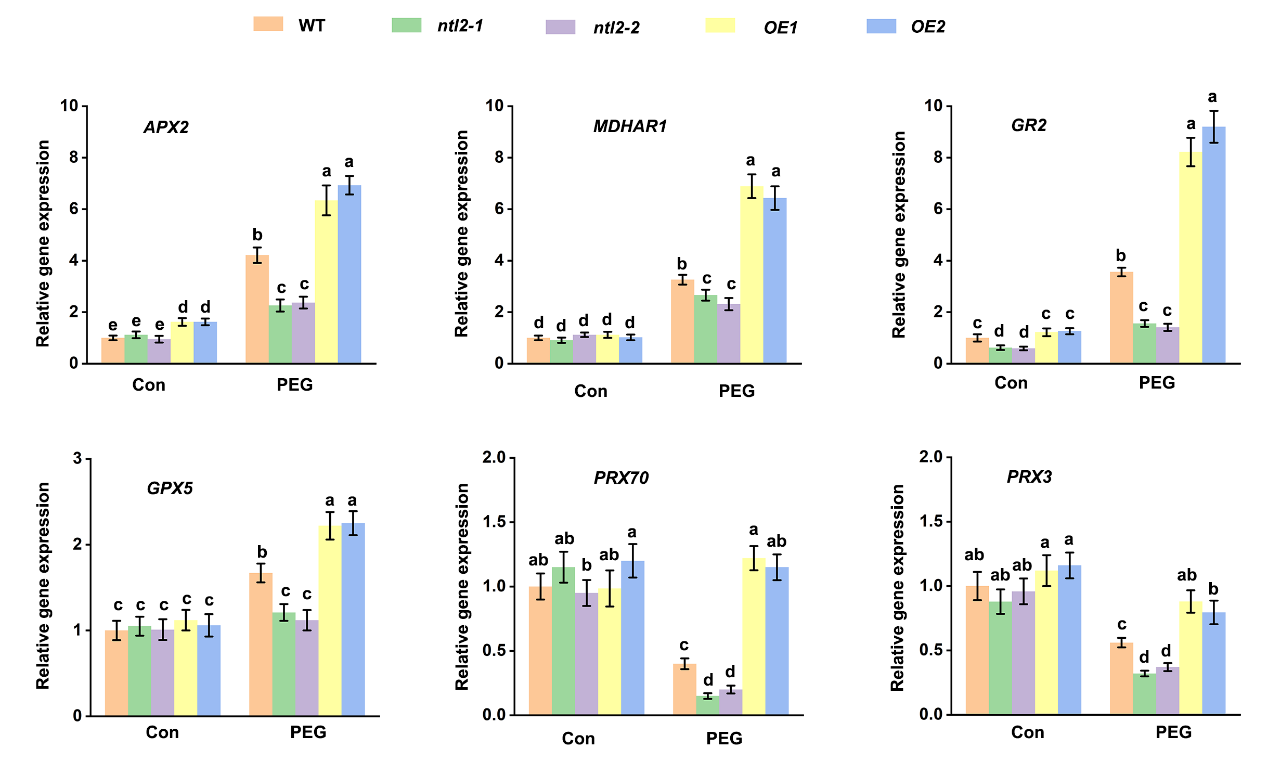
**

**Figure S7. OsNTL2 regulates the expression of gene encoding antioxidant enzymes in response to osmotic stress.** *OsACTIN* and *OsUBQ* was used as an internal control. Data are presented as mean ± SD (n = 3). Different letters denote significant differences at *p* < 0.05 (one-way ANOVA, Duncan’s multiple range test).

**Figure S8**


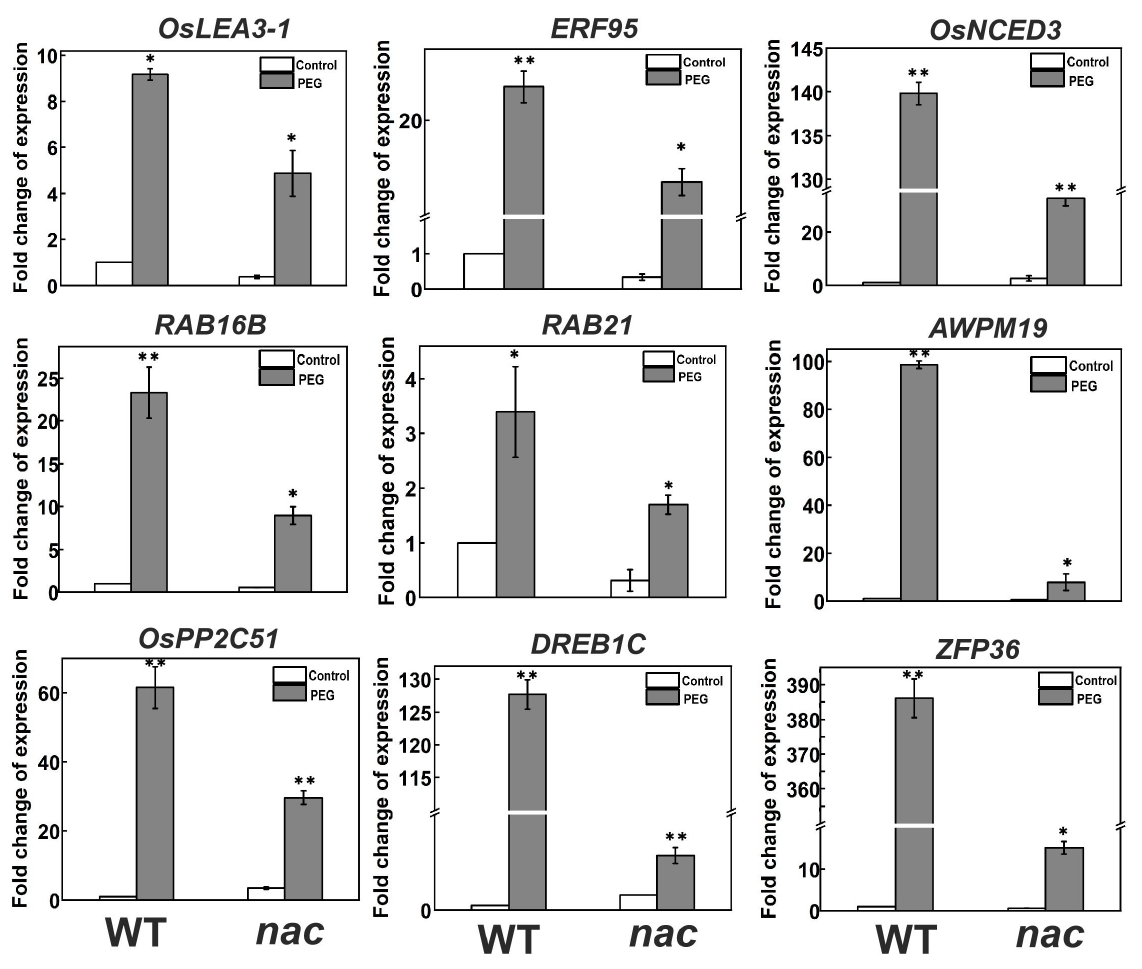

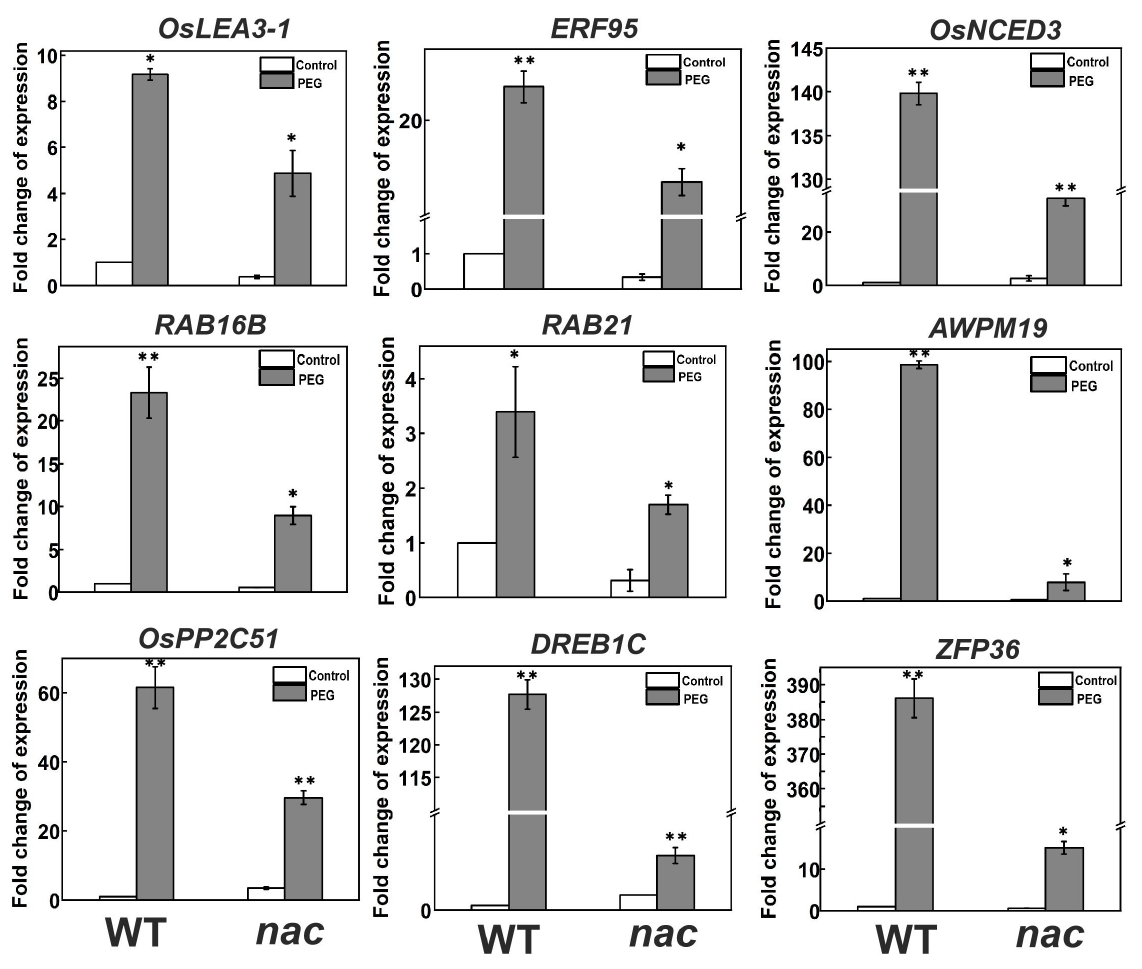


***ntl2-1***

**WT**

**WT**

***ntl2-1***

***ntl2-1***

**WT**

**Figure S8. Verification of gene expression by RT-qPCR.** *OsACTIN* and *OsUBQ* was used as an internal control. Asterisks indicate significant differences (***P* < 0.01, ****P* < 0.001; Student’s t-test).

**Figure S9**


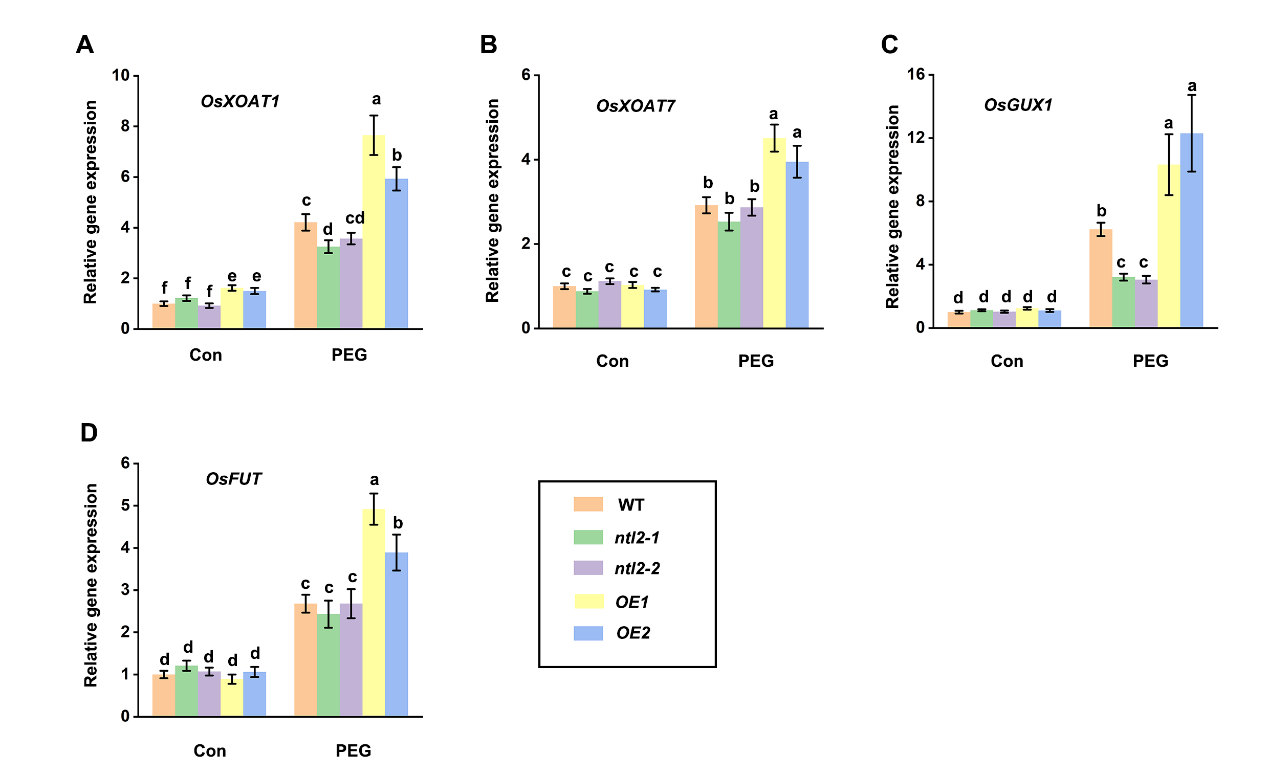


**Figure S9. Relative expression of genes involved in xylan biosynthesis in *ntl2* mutant, *OsNTL2*-OE, and WT plants under control and osmotic stress condition.** Fourteen-day-old rice seedlings were subjected to 20% PEG 6000 treatment for 24 h, and related gene expression were determined. Relative gene expression is the gene expression level of plants with treatment divided by that of plants before treatment, both of which were normalized to the expression of *OsACTIN* and *OsUBQ*. Data are presented as mean ± SD (n = 3).
